# Label‐Free Leukocyte Biophysical Profiling Using Impedance‐Deformability Cytometry for Rapid Cardiovascular Risk Stratification

**DOI:** 10.1002/advs.202516021

**Published:** 2025-11-28

**Authors:** Linwei He, Hui Min Tay, Feng Chen, Hong Sheng Cheng, Liang De Wang, Qiqi Nam, Arunachalam Thannirmalai, Lingyan Gong, Aram J. Chung, Nguan Soon Tan, King Ho Holden Li, Rinkoo Dalan, Siu Ling Wong, Han Wei Hou

**Affiliations:** ^1^ School of Mechanical and Aerospace Engineering Nanyang Technological University 50 Nanyang Avenue Singapore 639798 Singapore; ^2^ Lee Kong Chian School of Medicine Nanyang Technological University Mandalay Rd Singapore 308232 Singapore; ^3^ School of Biomedical Engineering Korea University 145 Anam‐ro, Seongbuk District Seoul 02841 Republic of Korea; ^4^ School of Biological Sciences Nanyang Technological University 60 Nanyang Dr Singapore 637551 Singapore; ^5^ Endocrinology Department Tan Tock Seng Hospital 11 Jln Tan Tock Seng Singapore 308433 Singapore; ^6^ Tan Tock Seng Hospital National Healthcare Group 11 Jln Tan Tock Seng Singapore 308433 Singapore

**Keywords:** biophysical profiling, diabetes, impedance cytometry, microfluidics, translational diagnostics

## Abstract

Type 2 diabetes mellitus (T2DM) presents a global health burden with cardiovascular disease (CVD) as its leading cause of mortality. A rapid clinical‐adaptable microfluidic workflow for label‐free CVD risk profiling based on neutrophil biophysical abnormalities is developed. This high‐throughput single cell (>1,000 cells min^−1^) “electro‐mechano‐phenotyping” method integrates Uniform Manifold Approximation and Projection analysis to assess leukocyte biophysical changes (size, deformability, and impedance properties) linked to inflammation, thrombosis, and hyperglycemia in vitro, and in diabetic and diabetic atherosclerosis‐prone mouse models. In a clinical study of healthy, pre‐diabetes, diabetes, and diabetic patients with CVD (DM‐CVD) subjects (*n* = 10–11 per group), DM‐CVD neutrophils exhibited a distinct impedance signature and pro‐inflammatory transcriptomic profile marked by cytoskeletal dysregulation and altered RhoA signaling. Principal component analysis (area under the curve = 0.971) identifies individuals with vascular dysfunction exhibiting increased carotid intima‐media thickness and reduced reactive hyperemia index. These findings support impedance‐based neutrophil profiling as a promising, cost‐effective strategy for cardiovascular risk stratification in T2DM.

## Graphical abstract

This study presents an impedance‐based single‐cell profiling platform that quantifies the electrical and mechanical properties of neutrophils across in vitro, in vivo, and clinical samples. The approach reveals distinct biophysical alterations associated with type 2 diabetes (T2DM) and cardiovascular complications, suggesting its potential utility for functional, label‐free assessment of immune dysregulation and cardiovascular risk stratification in T2DM.

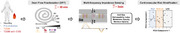

## 1 Introduction

Diabetes affects over 500 million people globally, posing a major healthcare challenge^[^ ^1^ ^]^ with cardiovascular disease (CVD) being the leading cause of death among individuals with type 2 diabetes mellitus (T2DM).^[^ ^2^ ^]^ As T2DM onset occurs at increasingly younger ages, patients will experience prolonged metabolic dysfunction and an elevated risk of early‐onset vascular complications,^[^ ^3^, ^4^, ^5^, ^6^ ^]^ necessitating a paradigm shift from traditional glucose‐centric management toward holistic strategies for early detection and prevention of subclinical atherosclerosis.^[^ ^7^ ^]^ CVD risk scores such as Framingham Risk Score use traditional risk factors, including age, gender, blood pressure, lipid profile, and smoking status, but they often overlook younger asymptomatic individuals with subclinical atherosclerosis.^[^ ^8^, ^9^, ^10^ ^]^ With recent clinical evidence suggesting a higher likelihood of regression in early stages of the disease,^[^ ^11^ ^]^ there is an unmet need to develop novel assays for monitoring cardiovascular health and risk stratification.^[^ ^12^ ^,^ ^13^ ^]^

Chronic low‐grade inflammation and persistent hyperglycemia are key drivers of immune dysfunction in diabetes,^[^ ^14^, ^15^ ^]^ and neutrophils, a primary effector of innate immunity, play critical roles in the pathogenesis of T2DM and CVD.^[^ ^16^, ^17^ ^]^ Mechanistically, neutrophils promote vascular damage through enhanced formation of neutrophil extracellular traps^[^ ^18^, ^19^ ^]^ and impaired cellular trafficking.^[^ ^20^ ^]^ Recent evidence further implicates neutrophil dysregulation with increased leukocyte adhesion, aberrant migration, and foam cell formation in endothelial dysfunction^[^ ^21^ ^]^ and atherosclerosis.^[^ ^22^, ^23^, ^24^ ^]^ However, current neutrophil phenotyping is hindered by labor‐intensive isolation protocols and costly antibody‐dependent immunoassays.

Biophysical intrinsic cellular properties such as size, stiffness^[^ ^25^, ^26^ ^]^ and electrical impedance^[^ ^27^ ^]^ are linked to immune dysfunction in diseases.^[^ ^28^, ^29^ ^]^ Microfluidic technologies employing hydrodynamic forces,^[^ ^30^, ^31^ ^]^ deterministic lateral flow displacement,^[^ ^32^ ^]^ constricted channels^[^ ^33^, ^34^ ^]^ and impedance cytometry^[^ ^35^, ^36^, ^37^ ^]^ enable rapid label‐free blood analysis.^[^ ^38^ ^]^ Nevertheless, the link between neutrophil biophysical abnormalities and diabetes or CVD remains poorly understood, representing a critical knowledge gap that hinders the clinical translation of these promising technologies.

Here, we present a rapid (< 40 min) clinical‐friendly integrated microfluidic workflow combining Dean Flow Fractionation (DFF) and impedance‐deformability cytometry for label‐free neutrophil isolation and biophysical characterization from small‐volume blood samples (< 200 µL). This unique “optics‐free” high throughput (> 1000 cells min^−1^) electrical screening of leukocyte size, deformability, membrane, and nucleus impedance enabled identification of distinct neutrophil biophysical signature under hyperglycemia, pro‐inflammatory (tumor necrosis factor alpha (TNF‐α)), and pro‐thrombotic (thrombin receptor acivator peptide 6 (TRAP‐6)) conditions in vitro. Validation using type 2 diabetes (*db/db*) and diabetic atherosclerosis‐prone (streptozotocin (*STZ*)‐induced apolipoprotein‐E (*apoE*)‐deficient) *(ApoE KO STZ)* mouse models revealed longitudinal alterations in neutrophil biophysical properties that correlated with diabetes progression and atherosclerotic severity. Clinical analysis of leukocytes from healthy, pre‐diabetes (Pre‐DM), diabetes (DM), and diabetes with cardiovascular complications (DM‐CVD) subjects (*n* = 10–11 per group) demonstrated progressive neutrophil biophysical changes, effectively visualized by Uniform Manifold Approximation and Projection (UMAP) clustering analysis. Transcriptomic analysis further confirmed a pro‐inflammatory phenotype in DM‐CVD neutrophils, highlighting dysregulated cytoskeletal regulation and the RhoA signaling pathway. Lastly, principal component analysis (PCA) integrating multiple leukocyte impedance parameters (≈27 impedance parameters) accurately identified (area under the curve (AUC) of 0.971) individuals with vascular dysfunction exhibiting increased carotid intima‐media thickness (CIMT) and reduced reactive hyperemia index. Collectively, the developed “electro‐mechano‐phenotyping” platform provides a rapid, cost‐effective approach to bridging the gap in cardiovascular risk stratification via neutrophil biophysical profiling.

## 2 Results

### 2.1 Microfluidic Workflow for Rapid Single‐Leukocyte Electro‐Mechano‐Phenotyping Using Impedance‐Deformability Cytometry

**Figure 1:**
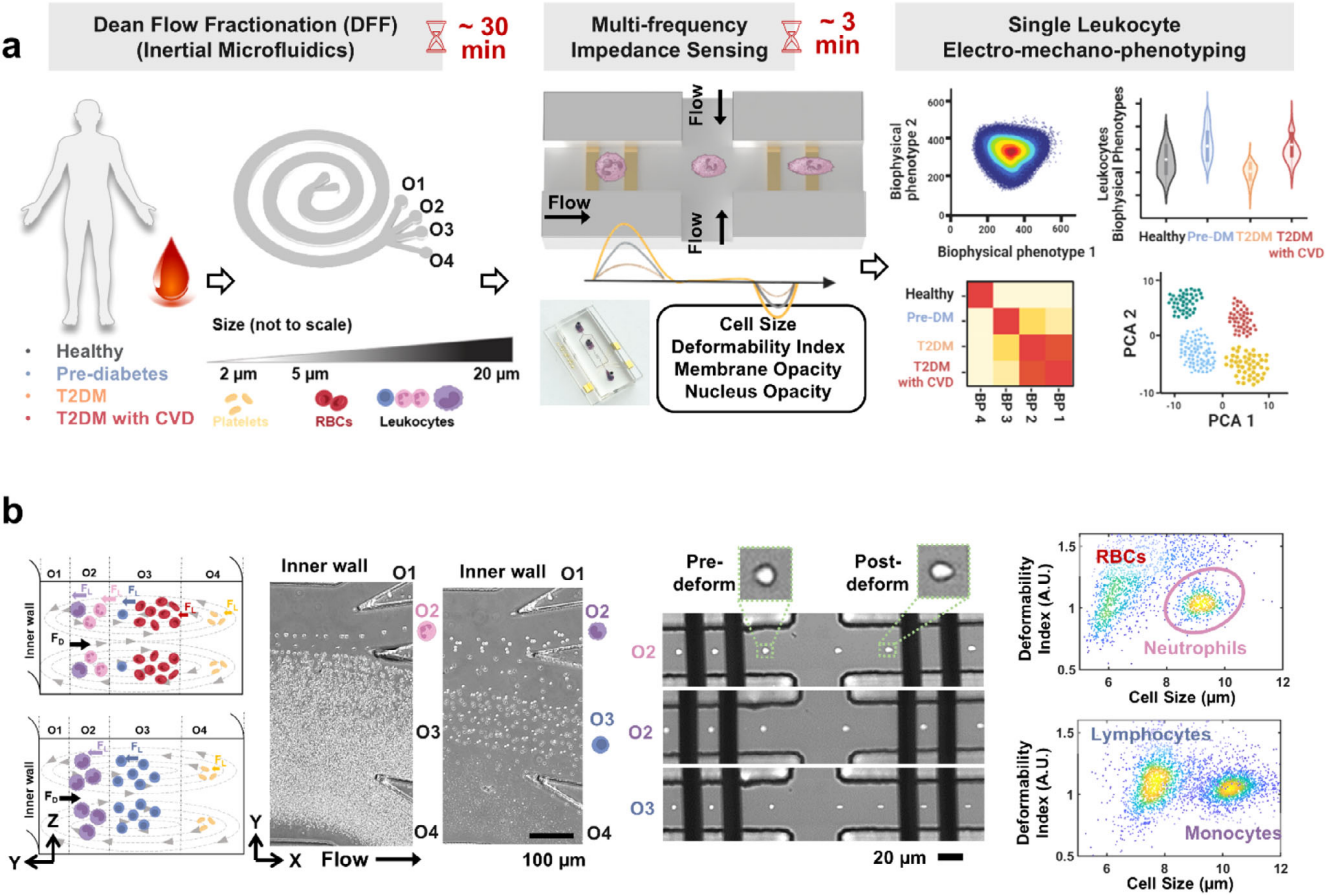
Microfluidic workflow for rapid single leukocyte electro‐mechano‐phenotyping using impedance‐deformability cytometry. a) Schematic illustration of leukocyte separation by Dean Flow Fractionation (DFF) and biophysical profiling (electro‐mechano‐phenotyping) using impedance‐deformability cytometry to study leukocyte biophysical properties at high throughput (1000 cells min^−1^) in healthy, pre‐diabetes (Pre‐DM), diabetes (DM), and diabetes with a history of cardiovascular complications (DM‐CVD) subjects (*n* = 10–11 per group). b) High‐speed imaging of size‐based neutrophil isolation (outlet 2, O2) from diluted whole blood, and peripheral blood mononuclear cells (PBMCs) fractionation into monocytes (O2) and lymphocytes (outlet 3, O3). Single‐cell viscoelastic stretching and dual impedance measurement of DFF‐sorted leukocytes at native and deformed states at three excitation frequencies (0.3, 1.72, and 12 MHz). Representative 2D impedance scatter plots with distinct identification of different leukocyte subtypes.

We developed a complete label‐free microfluidic workflow using two devices to facilitate rapid and gentle leukocyte isolation via DFF^[^ ^39^, ^40^ ^]^ and single‐cell biophysical profiling through impedance‐deformability cytometry^[^ ^26^, ^41^ ^]^ (**Figure** **1**a). Briefly, using multi‐frequency excitation signals (0.3, 1.72, and 12 MHz), we performed high‐throughput dual impedance measurements of single cells in their native and deformed state based on viscoelastic cell stretching at a cross‐flow junction (Figure 1b).^[^ ^26^ ^]^ This approach enables the capture of key biophysical attributes, including cell size, deformability, membrane opacity, and nucleus opacity, and UMAP was applied to map single‐leukocyte impedance signatures (see methods for impedance quantification). To validate the impedance setup, human leukemia cell line (HL‐60) was treated with latrunculin B^[^ ^42^ ^]^ (actin polymerization inhibitor) and blebbistatin^[^ ^43^ ^]^ (myosin II ATPase inhibitor), both of which exhibited a decrease in the deformability index (Figure S1, Supporting Information, n = 3). For clinical validation, healthy, Pre‐DM, DM, and DM‐CVD subjects were recruited (Table S1, Supporting Information) to assess the biophysical properties of various leukocyte subtypes (neutrophils, monocytes, lymphocytes). Larger neutrophils (≈10–12 µm) were directly isolated into outlet 2 (O2) of a 4‐outlet DFF device from diluted whole blood (1:10) with an efficiency of 75.1% ± 3.32% as determined by flow cytometry (Figure S2, Supporting Information, *n* = 5). Peripheral blood mononuclear cells (PBMC) obtained via Ficoll centrifugation were size‐fractionated by DFF into larger monocytes (O2) and smaller lymphocytes (outlet 3, O3) with cell purities of 66.8% ± 13.1% and 95.8% ± 4.24%, respectively (Figures 1b; S2, Supporting Information, *n* = 4). Residual red blood cells (RBCs) of DFF‐sorted neutrophil fractions were easily identified in 2D impedance scatter plots, thereby providing up to 12 biophysical‐related leukocyte impedance measurements per subject (Figure S3, Supporting Information).

### 2.2 Impedance‐Based Biophysical Profiling of Neutrophils under Hyperglycemia, Pro‐Inflammatory, and Pro‐Thrombotic In Vitro Conditions

**Figure 2:**
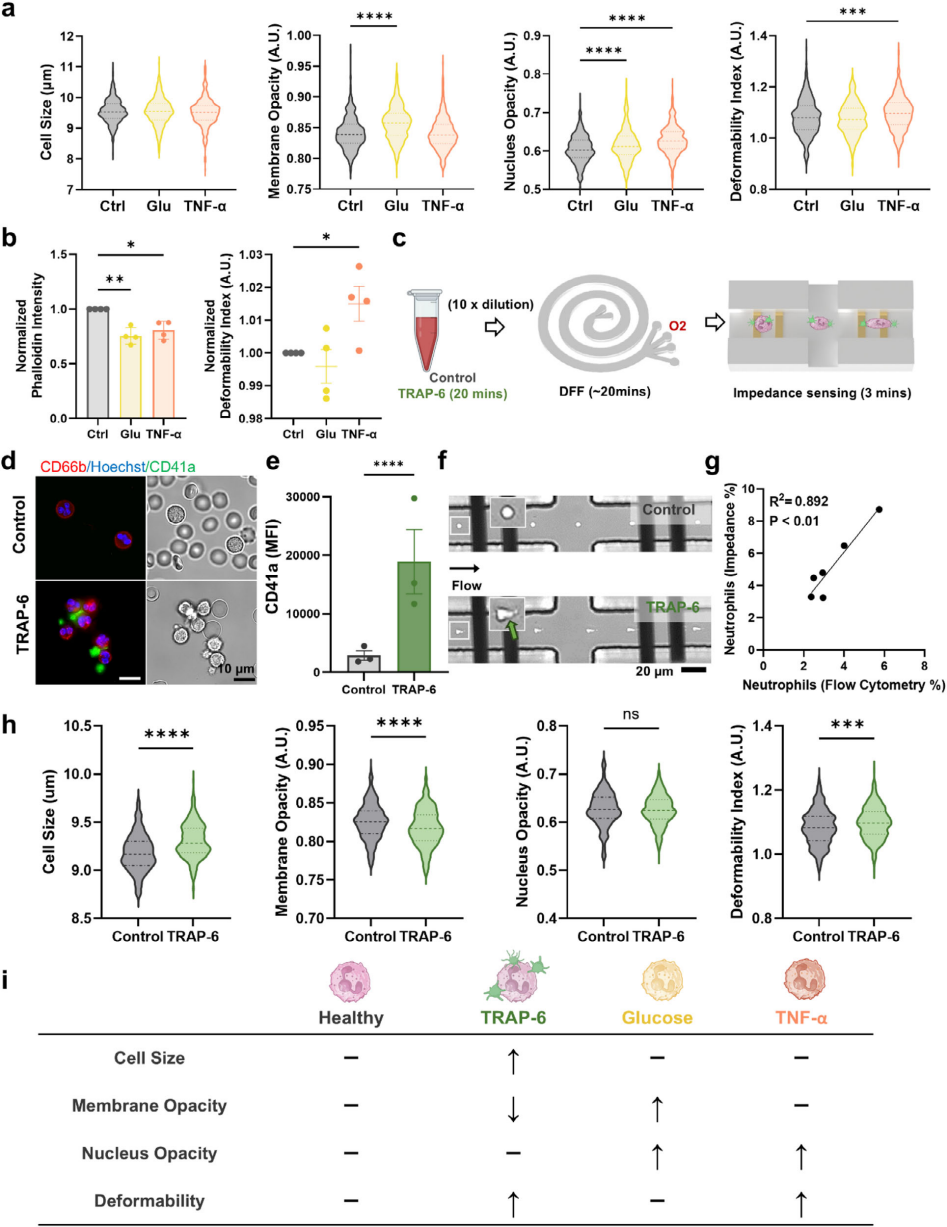
Impedance‐based biophysical profiling of neutrophils under hyperglycemia, pro‐inflammatory, and pro‐thrombotic in vitro conditions. a) Violin plots of neutrophil size, membrane opacity, nucleus opacity, and deformability index in control (Ctrl), high glucose [30 mm] (Glu), and pro‐inflammatory (tumor necrosis factor alpha, TNF‐α) conditions (500 cells (randomly selected) per sample, *n* = 4). Mean, and lower/upper quartiles are indicated as dotted lines. b) Normalized mean phalloidin intensity and mean deformability index for Ctrl, Glu, and TNF‐α‐treated neutrophils. c) Neutrophil isolation using DFF from diluted whole blood treated with platelet agonist thrombin receptor activator peptide 6 (TRAP‐6). d) Brightfield and fluorescence images indicating formation of neutrophil‐platelet aggregates (NPA) (CD66b^+^ (red), Hoechst^+^ (blue), and CD41a^+^ (green)) in TRAP‐6 treated blood. e) Mean platelet (CD41a) fluorescence intensity in NPAs (CD66b^+^CD41a^+^) (*n* = 3). f) High‐speed images of control neutrophils and TRAP‐6‐treated neutrophils in the impedance‐deformability device (green arrow indicates platelet binding on neutrophils). g) Correlation of neutrophils detected by flow cytometry (CD66b^+^) and impedance cytometry (*n* = 6). h) Violin plots of different biophysical properties for control and TRAP‐6‐treated neutrophils (500 cells (randomly selected) per sample, *n* = 3). i) Summary of impedance‐based neutrophil biophysical changes under different stimuli. ^*^ *P* < 0.05, ^**^ *P* < 0.01, ^***^ *P* < 0.001, ^****^ *P* < 0.0001 based on student's *t*‐test.

To investigate changes in neutrophil biophysical properties under hyperglycemic and pro‐inflammatory conditions, healthy neutrophils were treated with high glucose (30 mm)^[^ ^44^ ^]^ and TNF‐α (0.1 ng mL^−1^)^[^ ^45^ ^]^ for 30 min prior to impedance measurement, as these concentrations are widely used to model hyperglycemia‐ and cytokine‐induced activation in vitro. 2D impedance scatter plots for neutrophil size, membrane opacity, nucleus opacity, and deformability index are presented in Figure S4 (Supporting Information). Violin plots generated from 500 randomly selected single cells from each replicate (**Figure** **2**a, *n* = 4) revealed a significant increase in neutrophil membrane and nucleus opacity following glucose treatment. In contrast, TNF‐α‐treated neutrophils exhibited increased nucleus opacity and cell deformability. To further investigate cytoskeletal changes, F‐actin expression of neutrophils (phalloidin staining) was quantified by flow cytometry. Both glucose and TNF‐α treatments led to a significant decrease in normalized mean phalloidin intensity (relative to respective control samples) (Figure 2b). Notably, impedance‐based biophysical profiling demonstrated increased normalized mean deformability index and nucleus opacity for TNF‐α‐treated neutrophils (Figures 2b; S5, Supporting Information).

Next, whole blood was treated with platelet agonist TRAP‐6 (20 µm) for 30 min to induce platelet activation and the formation of neutrophil‐platelet aggregates (NPAs).^[^ ^46^ ^]^ To minimize shear‐induced platelet activation caused by centrifugation, neutrophils were isolated using DFF for impedance measurements (Figure 2c). Fluorescence and brightfield images showed morphological changes in TRAP‐6 treated neutrophils characterized by a spikier appearance and a notable increase in platelet (CD41a^+^, green) binding to neutrophils (CD66b^+^, red) (Figure 2d). The presence of NPAs was further confirmed by flow cytometry with increased CD41a expression (*n* = 3) (Figure 2e), and high speed imaging (Figure 2f). Impedance gating was applied to exclude residual RBCs (Figure S6, Supporting Information) and showed good correlation (R^2^ = 0.892, *P* < 0.01) in neutrophil quantification (%) between flow cytometry (CD66b^+^) and impedance profiling (*n* = 6) (Figure 2g).

To evaluate the biophysical changes in TRAP‐6‐treated neutrophils, violin plots were generated from 500 single cells randomly selected from each replicate (Figure 2h). A significant increase in neutrophil size and deformability, along with a decrease in membrane opacity, was observed, while nucleus opacity remained unchanged. Similar results were also observed using neutrophils isolated using immunomagnetic negative selection (Figure S7, Supporting Information, *n* = 3), further confirming that DFF is a gentle and cost‐effective isolation method suitable for subsequent in vivo and clinical studies. Taken together, these findings clearly demonstrate distinct neutrophil morphological alterations in hyperglycemic, pro‐inflammatory, and pro‐thrombotic conditions (summarized in Figure 2i), thus highlighting the importance of multi‐parametric single‐cell biophysical profiling.

### 2.3 Neutrophil Biophysical Alterations in Diabetes and Atherosclerosis‐Prone Mouse Models

Following in vitro studies, we investigated temporal changes in neutrophil biophysical properties in *db/db* and *ApoE KO STZ* mice. The *db/db* mice carry a mutation in the leptin receptor that leads to hyperphagia, obesity, and insulin resistance, resembling key metabolic features of human T2DM.^[^ ^47^, ^48^ ^]^ *ApoE KO* mice are an established mouse model known to develop robust atherosclerosis because of hypercholesterolemia. By inducing them diabetic using *STZ*, the *ApoE KO STZ* mice serve as a representative model for diabetes‐associated atherosclerosis.^[^ ^49^, ^50^ ^]^

**Figure 3:**
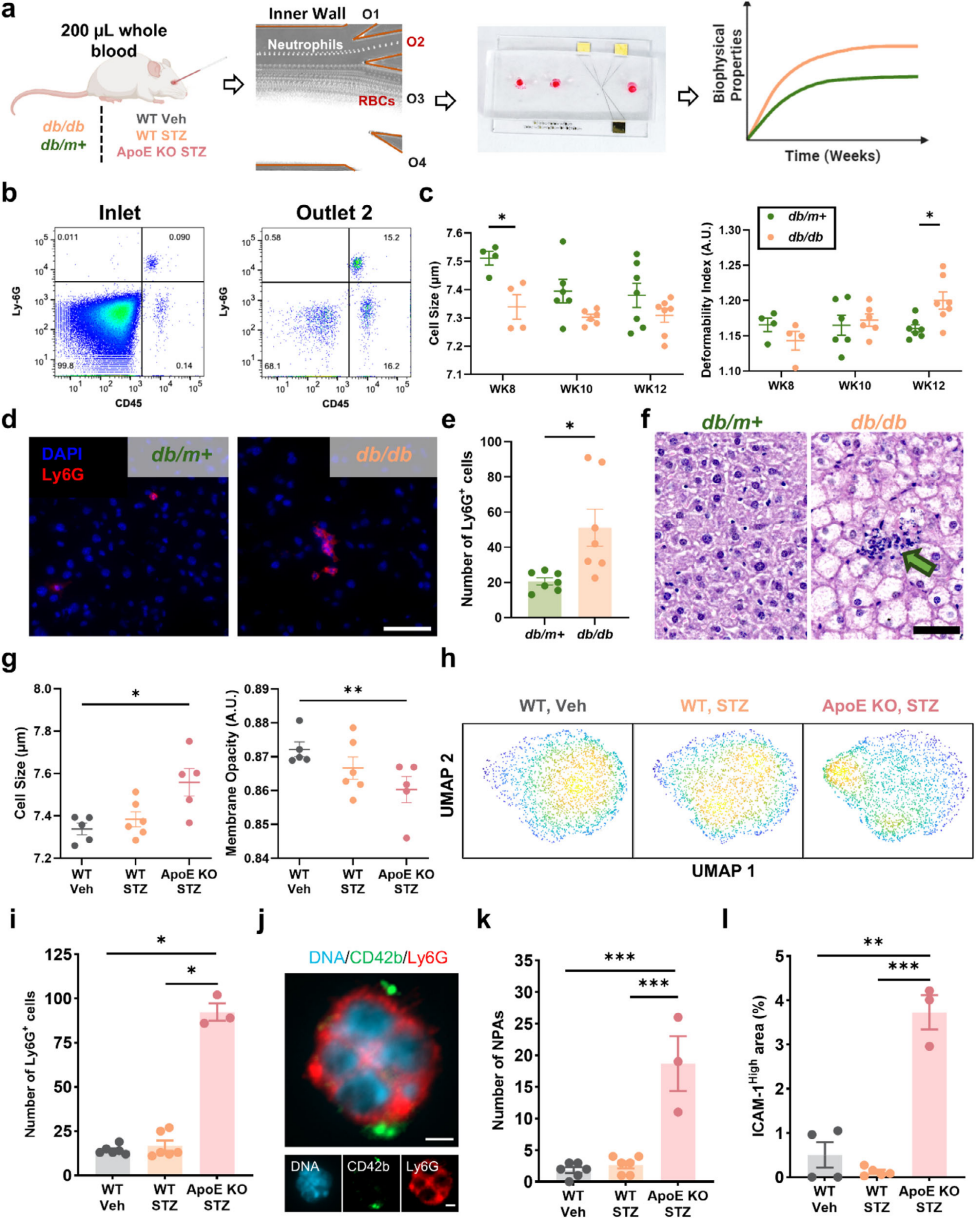
Neutrophil biophysical alterations in diabetes and diabetic atherosclerosis‐prone models. a) Longitudinal (biweekly) mouse neutrophil biophysical phenotyping using DFF and impedance‐deformability cytometry from 200 µL blood volume at each time point. b) Flow cytometry scatter plots (Ly6G and CD45) of diluted whole blood (inlet) and DFF‐sorted leukocytes (O2). c) Impedance‐based quantification of leukocyte cell size and deformability in diabetic (*db/db*) and non‐diabetic (*db/m+)* mice from week 8 to week 12. d) Immunofluorescence images showing neutrophil (Ly6G^+^ DAPI^+^) infiltration in mouse livers (Scale bar: 50 µm), e) Quantification of Ly6G^+^ cells from 11 high‐power fields, and f) H&E staining of *db/m+* and *db/db* mouse livers at week 12. Green arrow indicates immune cell infiltration (Scale bar: 50 µm). g) Cell size and membrane opacity, and h) uniform manifold approximation and projection (UMAP) analysis of 9 impedance parameters of DFF‐sorted leukocyte (O2) for wild type (*WT Veh*), streptozotocin (*STZ*)‐induced diabetic (*WT STZ*), and *STZ*‐induced diabetic atherosclerosis‐prone (apolipoprotein‐E (apoE)‐deficient) (*ApoE KO STZ*) mice at week 8 post diabetes induction. i) Quantification of Ly6G^+^ cells in mouse liver tissue from 11 high‐power fields. j) Immunofluorescence overlay images (Hoechst^+^, CD42b^+^, and Ly6G^+^ for nuclei, platelets, and neutrophils, respectively) showing NPAs in mouse liver (Scale bar: 2 µm). k) Quantification of NPAs in the mouse liver from 11 high‐power fields. l) Percentage of the area in mouse liver expressing high levels of ICAM‐1. ^*^ *P* < 0.05, ^**^ *P* < 0.01, ^***^ *P* < 0.001 based on Mann Whitney test (3i) or student's *t*‐test (others).

Small blood volumes (≈200 µL) were collected biweekly (week 8 to week 12) from *db/db* (diabetic) mice and *db/m+* (control) mice for neutrophil isolation using DFF to eliminate the need to sacrifice mice at each time point (**Figure** **3**a). DFF‐sorted leukocytes (O2) were enriched for neutrophils (Ly6G^+^CD45^+^) with significant RBCs removal (*n* = 4) (Figure 3b), allowing clear identification of leukocyte population from residual RBCs on impedance scatter plots (Figure S8, Supporting Information). Weekly measurements showed significantly higher body weight and blood glucose in *db/db* mice since 6 weeks of age, confirming progressive diabetes development in this animal model (Figure S9, Supporting Information).

Interestingly, leukocyte size decreased in both *db/m+* and *db/db* mice from 8 to week 10 with diabetic leukocytes consistently smaller than control leukocytes and an observed significant difference at week 8 (*P* < 0.05) (Figure 3c). Leukocytes from *db/db* mice displayed progressively higher deformability over time, which became significantly higher than control mice at week 12 (*P* < 0.05), suggesting an increased potential for diabetic leukocytes (smaller and more deformable) to infiltrate inflamed organs. While leukocyte membrane opacity remained relatively unchanged in both groups, leukocyte nucleus opacity increased significantly in *db/db* mice at week 12 (*P* < 0.05) (Figure S10, Supporting Information). An increase in liver weight was observed in *db/db* mice at week 12 (Figure S9, Supporting Information), suggesting hepatic alterations (such as fatty liver^[^ ^51^ ^]^) associated with obesity and diabetes.^[^ ^52^ ^]^ To further understand the pathophysiological impact of neutrophil biophysical abnormalities in diabetes,^[^ ^17^ ^]^ we quantified neutrophil infiltration in the mouse liver at week 12. Immunofluorescence staining revealed a significant increase in Ly6G^+^ neutrophils in the liver of *db/db* mice compared to *db/m+* mouse liver (*P* < 0.01, *n* = 7), based on (Figure 3d,e), which was also observed using hematoxylin and eosin (H&E) staining (Figure 3f).

Next, to determine if neutrophil biophysical differences are exacerbated in CVD, we isolated leukocytes by DFF from *ApoE KO STZ* mice for impedance analysis. Diabetic mice (*WT STZ*) and wild‐type controls (*WT Veh*) served as controls, with leukocytes isolated at week 8 post diabetes induction with *STZ*. Unlike *db/db* mice, DFF‐sorted leukocytes from *ApoE KO STZ* mice exhibited significant differences in cell size (*P* < 0.05) and membrane opacity (*P* < 0.01) compared to *WT Veh* (Figure 3g). While nucleus opacity remained unchanged across groups, the cell deformability index was elevated in *WT STZ* mice (Figure S11, Supporting Information) which was similar to that observed in *db/db* mice (Figure 3c). UMAP analysis, incorporating nine impedance‐measured biophysical parameters, revealed significant alterations in leukocyte biophysical properties and distinct leukocyte clustering in *ApoE KO STZ* mice (Figure 3h). Magnitude mapping indicated that these changes were primarily driven by increased cell size and reduced membrane opacity (Figure S12, Supporting Information). Furthermore, Ly6G^+^ neutrophil infiltration into the livers of *ApoE KO STZ* mice was significantly higher (≈4–5 fold, *P* < 0.05) compared to *WT STZ and WT Veh* (*P* < 0.05) (Figures 3i; S13, Supporting Information). NPAs (Ly6G^+^CD42b^+^) from 11 high‐power fields of liver sections were markedly increased in *ApoE KO STZ* mice (Figure 3j,k). Finally, ICAM‐1 expression in the livers of *ApoE KO STZ* mice was ≈7‐fold higher compared to *WT Veh* and ≈30‐fold increase compared to *WT STZ*, suggesting severe vascular inflammation (Figures 3l; S14, Supporting Information). Taken together, these findings indicate significant temporal neutrophil biophysical alterations in diabetes mice, which were more pronounced in the *ApoE KO STZ* mice, suggesting their potential as early biomarkers of CVD risk stratification in diabetes.

### 2.4 Metabolic Profile, Blood Cell Count, and Biomarkers of Subclinical Atherosclerosis in Healthy, Pre‐DM, DM, and DM‐CVD Subjects

**Figure 4:**
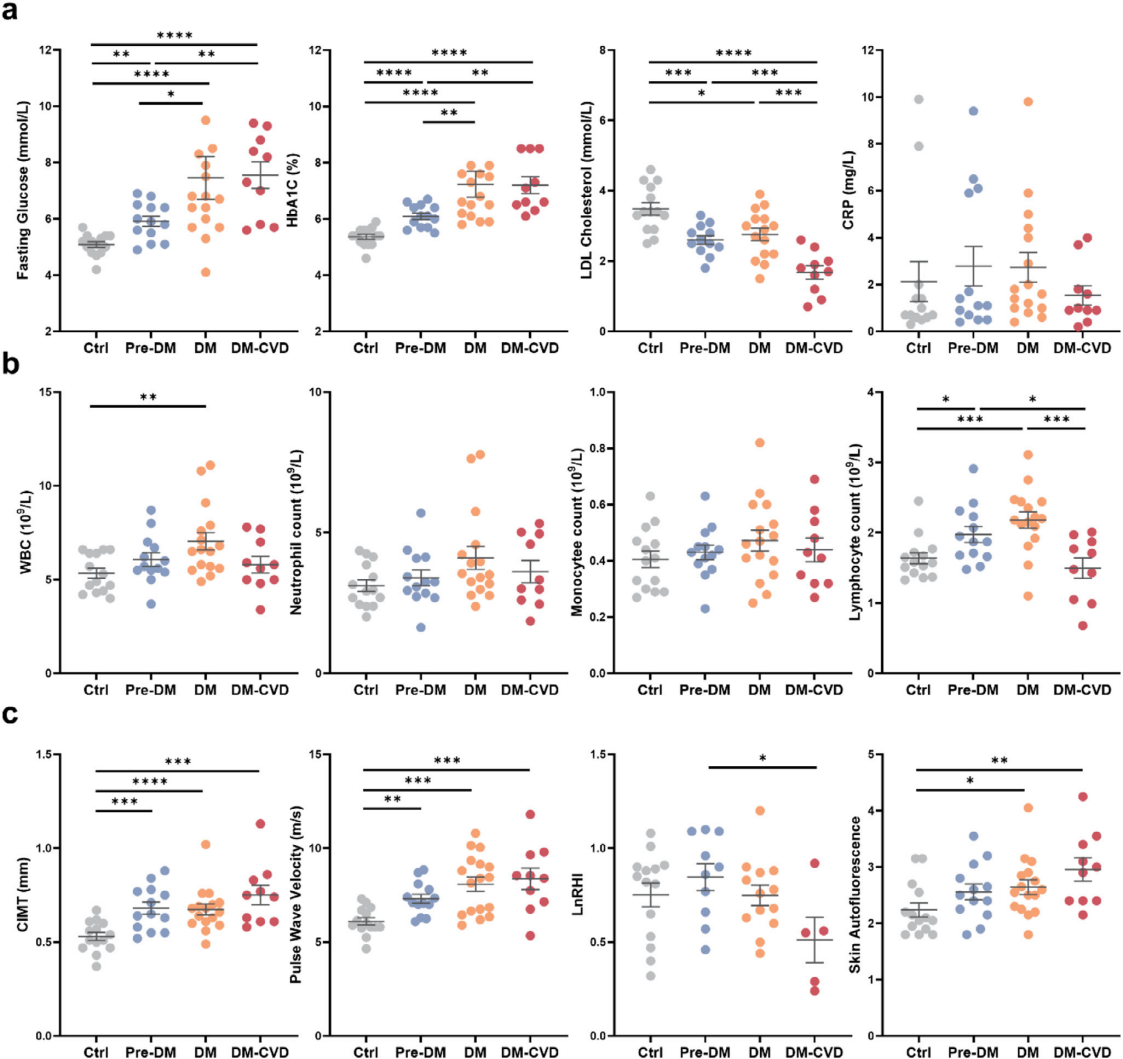
Metabolic profile, blood cell count, and biomarkers of subclinical atherosclerosis in healthy, Pre‐DM, DM, and DM‐CVD subjects. a) Blood fasting glucose, hemoglobin A1C (HbA1c), low‐density lipoprotein (LDL) cholesterol, and C‐reactive protein (CRP). b) Total white blood cells (WBC), neutrophils, monocytes, and lymphocytes count. c) Carotid intima‐media thickness (CIMT), pulse wave velocity (PWV), natural logarithmic transformed reactive hyperemia‐peripheral arterial tonometry index (LnRHI), and skin autofluorescence (SAF) in healthy controls (Ctrl, *n* = 14), pre‐diabetes (Pre‐DM, *n* = 13), Type 2 diabetes mellitus (DM, *n* = 16), and T2DM with history of cardiovascular complications (DM‐CVD, *n* = 10) subjects. ^*^ *P* < 0.05, ^**^ *P* < 0.01, ^***^ *P* < 0.001 based on student's *t*‐test.

With growing evidence indicating leukocyte implication in T2DM pathogenesis and atherosclerosis,^[^ ^53^ ^]^ we recruited 4 different subject groups including healthy controls (Ctrl, *n* = 14), pre‐diabetes (Pre‐DM, *n* = 13), T2DM patients without CVD complications (DM, *n* = 16), and T2DM patients with history of CVD complications (DM‐CVD, *n* = 10) based on the recruitment criteria in Table S1 (Supporting Information). Key clinical metabolic, blood, and endothelial function test parameters are summarized in Table S2 (Supporting Information). Fasting glucose and hemoglobin A1C (HbA1c) levels progressively increased with disease severity, with DM and DM‐CVD subjects exhibiting poor glycemic control (*P* < 0.0001) as compared to Ctrl and Pre‐DM groups (**Figure** **4**a). Low‐density lipoprotein (LDL) cholesterol levels were lowest in DM‐CVD subjects, likely due to statin therapy for lipid control. Sensitive C‐reactive protein (CRP), a key marker of inflammation, exhibited high interindividual variability and showed no significant differences across groups. Notably, while leukocyte count, a standard clinical parameter for assessing inflammation,^[^ ^54^ ^]^ was the highest in the DM group, neutrophil and monocyte counts remained comparable across groups (Figure 4b). Lymphocyte counts were highest in the DM group, while DM‐CVD subjects had significantly lower lymphocytes (*P* < 0.0001).

CIMT, a surrogate marker of subclinical atherosclerosis,^[^ ^55^ ^]^ increased with disease severity (0.53 ± 0.08 mm (Ctrl), 0.68 ± 0.12 mm (Pre‐DM), 0.67 ± 0.12 mm (DM), and 0.75 ± 0.17 mm (DM‐CVD)) (Figure 4c). A similar trend was observed for pulse wave velocity (PWV), a widely used index of arterial wall stiffness for non‐invasive assessment of early atherosclerosis (6.07 ± 0.72 m s^−1^ (Ctrl), 7.31 ± 0.87 m s^−1^ (Pre‐DM), 8.17 ± 1.58 m s^−1^ (DM), and 8.3 ± 1.80 m s^−1^ (DM‐CVD)), indicating elevated vascular dysfunction in DM and DM‐CVD subjects.^[^ ^56^ ^]^ The natural logarithmic‐transformed reactive hyperemia‐peripheral arterial tonometry index (LnRHI), which assesses endothelial function and cardiovascular health,^[^ ^57^ ^]^ exhibited a declining trend with disease severity (0.75 ± 0.24 (Ctrl), 0.85 ± 0.23 (Pre‐DM), 0.75 ± 0.2 (DM), and 0.51 ± 0.27 (DM‐CVD)), indicating impaired endothelial function in DM‐CVD subjects. Skin autofluorescence (SAF), a measure of advanced glycation end‐product accumulation,^[^ ^58^ ^]^ also increased with disease severity (2.31 ± 0.5 (Ctrl), 2.56 ± 0.5 (Pre‐DM), 2.64 ± 0.52 (DM), and 2.96 ± 0.65 (DM‐CVD)), suggesting increased oxidative stress and inflammation. Taken together, these results confirmed that DM‐CVD subjects exhibited compromised vascular health based on subclinical atherosclerosis measurements despite showing no significant differences in baseline inflammation as indicated by CRP and blood cell count.

### 2.5 Impedance‐Based Neutrophil Biophysical Profiling in Clinical Subjects

**Figure 5:**
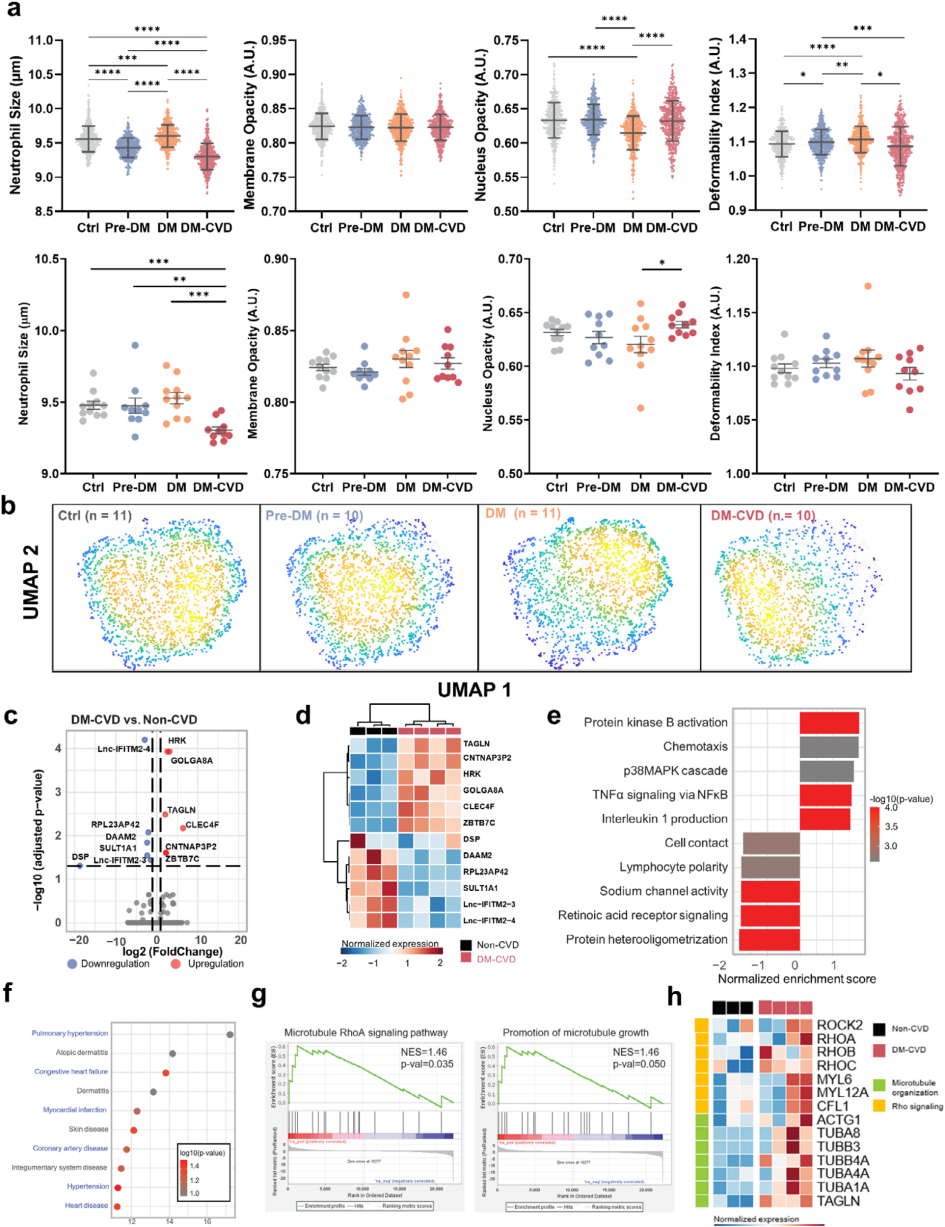
Impedance‐based neutrophil biophysical profiling in clinical subjects. a) (top) Violin plots of cell population (500 cells were randomly selected for each subject) and (lower) dot plots of individual mean values of neutrophil size, membrane opacity, nucleus opacity, and deformability index in healthy controls (Ctrl, *n* = 11), pre‐diabetes (Pre‐DM, *n* = 10), Type 2 diabetes mellitus (DM, *n* = 11), and diabetes with history of cardiovascular complications (DM‐CVD, *n* = 10). b) UMAP analysis of impedance‐based neutrophil biophysical properties (total 9 parameters) indicates distinct clustering of DM‐CVD neutrophils. c) Volcano plot and d) heatmap showing the differentially expressed genes (DEGs) between DM‐CVD or non‐CVD groups. e) Top activation and inhibited gene sets in DM‐CVD compared to non‐CVD groups. NES = normalized enrichment score. f) Top enriched disease ontologies based on differential transcriptomes comparing DM‐CVD to non‐CVD groups. g) Targeted GSEA showing the activation of microtubule remodeling and RhoA signaling in DM‐CVD neutrophils and h) the corresponding upregulated genes. ^*^ *P* < 0.05, ^**^ *P* < 0.01, ^***^ *P* < 0.001, ^****^ *P* < 0.0001 based on student's *t*‐test.

As blood cell counts remain inadequate for assessing endothelial dysfunction, we performed impedance‐based biophysical profiling of neutrophils, monocytes, and lymphocytes isolated using DFF from Ctrl (*n* = 11), Pre‐DM (*n* = 10), DM (*n* = 11), and DM‐CVD (*n* = 10) subjects. Violin plots of various biophysical properties for neutrophils (**Figure** **5**a), lymphocytes (Figure S15, Supporting Information), and monocytes (Figure S16, Supporting Information) were generated by randomly selecting 500 single‐cell data points from each subject. A significant decrease in neutrophil size was observed in DM‐CVD as compared to DM subjects (*P* < 0.0001). While membrane opacity did not differ significantly among the groups, nucleus opacity was higher in DM‐CVD neutrophils than in DM neutrophils (*P* < 0.0001). Additionally, the deformability index of DM‐CVD neutrophils was lower than DM neutrophils (*P* < 0.05), indicating substantial changes in neutrophil biophysical properties in DM‐CVD subjects. Analysis of mean neutrophil biophysical parameters per subject revealed similar trends, with DM‐CVD neutrophils being smaller and exhibiting higher nucleus opacity than DM neutrophils. Although DM‐CVD neutrophils were stiffer than DM neutrophils, this difference was not statistically significant, which may reflect the inherent neutrophil biophysical heterogeneity among patient samples. Interestingly, UMAP analysis of all 9 impedance‐derived biophysical parameters showed distinct clustering of DM‐CVD and DM neutrophils (Figure 5b). Magnitude mapping confirmed that these changes were driven by a combination of electrical and mechanical properties, including cell size, deformability, and nucleus opacity (Figure S17, Supporting Information). Notably, this clinical UMAP neutrophil biophysical analysis was consistent with in vivo results of *STZ‐induced apoE‐deficient* mice, strongly suggesting that neutrophil dysfunction and their biophysical abnormalities play significant roles in diabetes and CVD. Biophysical changes were also observed in DM‐CVD lymphocytes and monocytes, including increased lymphocyte deformability and higher nuclear opacity in monocytes. However, UMAP analysis revealed greater overlap between lymphocyte and monocyte populations in DM and DM‐CVD subjects (Figures S15 and S16, Supporting Information), which suggests that neutrophils may play a more prominent role in CVD pathophysiology.

To further characterize DM‐CVD neutrophils, we performed transcriptomics analysis (bulk‐RNA sequencing) of neutrophils from DM‐CVD (*n* = 3) and non‐CVD subjects (*n* = 4, Pre‐DM/DM). A panel of 12 differentially expressed genes (DEGs) in DM‐CVD neutrophils, with six genes upregulated and six downregulated as compared to non‐CVD neutrophils, was identified (Figure 5c,d). Functionally, neutrophils from DM‐CVD patients exhibited increased pro‐inflammatory activity, as evidenced by the activation of the p38 MAPK signaling pathway, the NF‐κB signaling cascade, and elevated interleukin production (Figure 5e). Disease ontology enrichment analysis revealed a strong association between the neutrophil transcriptomes and various cardiovascular diseases, underscoring their crucial role in CVD pathogenesis (Figure 5f). Additionally, several DEGs, such as *TAGLN*, *GOLGA8A*, *DSP*, and *DAAM2*, were linked to structural changes and cytoskeletal regulation (Figure 5d). Targeted functional analysis further indicated the activation of microtubule remodeling and RhoA signaling DM‐CVD neutrophils (Figure 5g,h). Collectively, these findings corroborate the biophysical changes observed in DM‐CVD neutrophils using impedance‐deformability cytometry, which are closely associated with elevated inflammation^[^ ^59^ ^]^ and structural modifications.^[^ ^60^ ^]^

### 2.6 Multi‐Parametric Leukocyte Biophysical Properties for Label‐Free Vascular Risk Stratification

**Figure 6:**
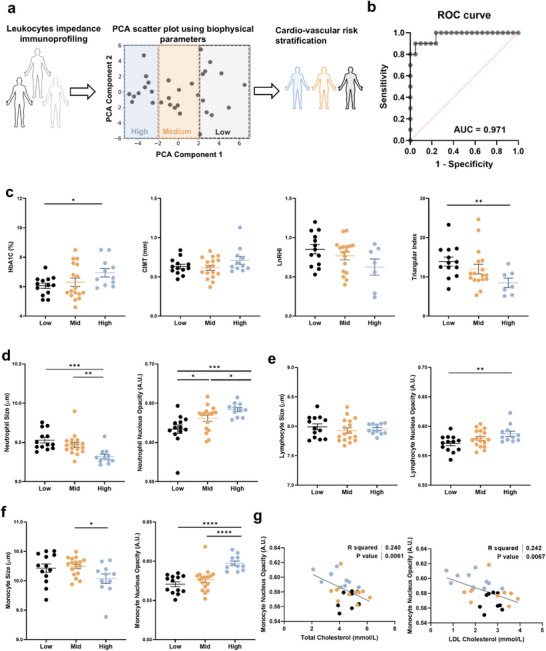
Principal component analysis of multi‐parametric leukocyte biophysical properties for label‐free vascular risk stratification. a) Impedance‐based vascular risk stratification into low‐risk (black), medium‐risk (orange), and high‐risk (blue) subjects based on PCA scores. b) Receiver operating characteristic (ROC) curve analysis for impedance‐classified cardiovascular risk factors in relation to patients’ clinical CVD history (CVD vs non‐CVD). Comparison of c) clinical parameters including HbA1c, CIMT, LnRHI, and heart rate variability triangular index, and d–f) impedance‐based biophysical parameters including cell size and nucleus opacity for neutrophils, lymphocytes, and monocytes for impedance‐classified low risk (*n* = 11), medium risk (*n* = 10), and high risk (*n* = 10) subjects. g) Correlations between monocyte nucleus opacity and clinical measurements of total cholesterol and LDL cholesterol. ^*^ *P* < 0.05, ^**^ *P* < 0.01, ^***^ *P* < 0.001, ^****^ *P* < 0.0001 based on student's *t*‐test.

Since various leukocyte subtypes exhibited distinct biophysical changes in DM‐CVD subjects, we combined all the leukocyte impedance parameters (27 parameters per subject) and performed PCA. Based on their PCA scores, subjects were classified into different vascular risk groups, including low‐risk (PCA component 1 > 2, Low), medium‐risk (‐2 <PCA component 1 < 2, Medium), and high‐risk (PCA component 1 < ‐2, High) subjects (**Figure** **6**a). This classification was determined by analyzing PCA scores in relation to key clinical parameters, including HbA1c, CIMT, LnRHI, and heart rate variability triangular index in Pre‐DM/DM/DM‐CVD subjects. Notably, most DM‐CVD subjects were clustered within the high‐risk group (PCA score <−2). Additionally, LDL cholesterol levels (*P* < 0.01) and SAF were negatively correlated to PCA score (*P* < 0.01) (Figure S18, Supporting Information). Using this classification, 9 out of 10 high‐risk subjects were DM‐CVD patients, while the remaining one was a pre‐DM subject who had higher PWV (clinical indicator of arterial stiffening) (8.85 m s^−1^) as compared to other pre‐DM individuals (7.31 ± 0.87 m s^−1^) (Table S2, Supporting Information). The receiver operating characteristic (ROC) curve of PCA scores against patients’ clinical history (CVD vs non‐CVD) yielded an AUC of 0.971 (Figure 6b), indicating a strong potential predictive potential for impedance‐derived PCA scores in vascular risk stratification among diabetic patients. Comparison of key clinical parameters among the 3 risk groups revealed significant differences in HbA1c (*P* < 0.05) and heart rate variability triangular index (*P* < 0.01) between high‐ and low‐risk subjects (Figure 6c). Higher CIMT and lower LnRHI were also observed in high‐risk subjects, indicating the presence of subclinical atherosclerosis.

Notably, several leukocyte biophysical parameters correlated with PCA‐based vascular risk severity. High‐risk subjects exhibited smaller neutrophil size and increased neutrophil nucleus opacity (Figure 6d), higher lymphocyte nucleus opacity (Figure 6e), and smaller monocyte size with increased nucleus opacity (Figure 6f), highlighting the importance of leukocyte dysfunction in diabetes‐related vascular complications. Furthermore, monocyte nucleus opacity showed significant correlation with total cholesterol (*P* < 0.01) and LDL cholesterol (*P* < 0.01) (Figure 6g), potentially reflecting an effect of monocyte cholesterol uptake on impedance measurements.^[^ ^37^ ^]^ Overall, these findings strongly support the potential of impedance‐based leukocyte biophysical profiling as a rapid, label‐free tool for vascular risk stratification in diabetes.

## 3 Discussion

In this study, we present a rapid, user‐friendly microfluidic workflow for label‐free neutrophil isolation and high throughput (1000 cells min^−1^) single‐cell biophysical profiling “electro‐mechano‐phenotyping”. Our platform enables simultaneous mechanical and electrical characterization of neutrophils from small blood volumes (≈200 µL), making it a scalable and minimally invasive tool for clinical applications. While previous biophysical studies primarily focus on single or two biophysical parameters (e.g., cell stiffness or electrical impedance),^[^ ^38^, ^42^ ^]^ our platform offers a multi‐frequency impedance approach to capture up to 9 impedance attributes per cell. This enabled us to uncover distinct electrical and mechanical signatures associated with pro‐inflammatory, hyperglycemic, and pro‐thrombotic conditions (Figure 2i). Indeed, we provide the first comprehensive demonstration of impedance‐based neutrophil electro‐mechano‐phenotyping across in vitro, in vivo, and clinical settings. By capturing multi‐parametric biophysical signatures of neutrophils, we revealed previously uncharacterized mechanical and electrical alterations associated with inflammation, diabetes, and CVD, and their correlations with subclinical atherosclerosis (CIMT, LnRHI). While established circulating biomarkers remain useful for cardiovascular risk assessment, they primarily reflect downstream inflammation.^[^ ^61^, ^62^ ^]^ In contrast, our impedance‐based leukocyte profiling offers a complementary functional approach that directly assesses immune‐cell activity and may detect early or subclinical inflammatory changes preceding conventional biomarker elevation.

A key advantage of inertial (DFF)‐based workflow is the ability to process small blood volumes, enabling longitudinal neutrophil analysis in the same mice without requiring sacrifice. This reduces the number of animals required per study and lowers experimental costs. In the *db/db* mouse model, we observed a progressive decrease in neutrophil size over time, which could be associated with cellular senescence (Figure 3c).^[^ ^63^ ^]^ We also detected increased cell deformability at week 12, aligning with our in vitro hyperglycemia and TNF‐α treatment, suggesting a link between chronic inflammation, hyperglycemia, and altered neutrophil mechanics. The increased neutrophil infiltration in the liver of *db/db* mice reinforces the hypothesis that more deformable neutrophils may have enhanced organ infiltration potential (Figure 3d–f). In the atherosclerosis‐prone *ApoE KO STZ* mouse model, larger and more deformable neutrophils with reduced membrane opacity resemble the TRAP‐6‐induced NPA phenotype (Figures 3g and 2h) with histological confirmation of increased NPAs in the livers (Figure 3k). These findings suggest that diabetes and atherosclerosis induce distinct neutrophil biophysical changes: chronic hyperglycemia promotes increased neutrophil deformability and organ infiltration, whereas platelet activation in CVD alters neutrophil size, membrane integrity, and mechanical properties. While both *db/db* and *ApoE KO STZ* models recapitulate key metabolic and vascular features of human diabetes and CVD, they each have inherent limitations. The *db/db* model represents a monogenic leptin‐receptor defect rather than the multifactorial etiology of human T2DM, and hyperglycemia in the *ApoE KO STZ* model is chemically induced rather than resulting from insulin resistance.

While leukocyte counts did not significantly differ among Ctrl, Pre‐DM, DM, and DM‐CVD subjects (Figure 4b), our multi‐parametric leukocyte impedance profiling showed significant biophysical alterations in DM‐CVD neutrophils. UMAP analysis indicated distinct clustering of DM‐CVD neutrophils, suggesting a unique biophysical signature linked to vascular complications (Figure 5b). Bulk RNA sequencing confirmed that DM‐CVD neutrophils were pro‐inflammatory with dysregulated genes involved in cytoskeletal remodeling and RhoA signaling (Figure 5c–h). Notably, alterations in neutrophil size and deformability observed in *ApoE KO STZ* mouse models and DM‐CVD patients were consistent, which suggests a potential link between neutrophil phenotype and cholesterol or LDL cholesterol levels (Figures 3, 4, and 5a), highlighting their relevance in atherosclerotic plaque formation and cardiovascular risk.^[^ ^64^ ^]^

It should be noted that intra‐patient neutrophil heterogeneity may contribute to the observed overlap between patient groups, and future research using single‐cell sequencing will help us better understand the molecular mechanisms underlying the observed biophysical changes in diabetic neutrophils, with a particular focus on cytoskeletal remodeling and oxidative stress. Given that neutrophil deformability and nucleus opacity alterations are associated with inflammatory and metabolic conditions, it is critical to investigate the signaling pathways and structural modifications driving these changes. Understanding how oxidative stress influences neutrophil mechanics and function could provide deeper insights into their role in diabetes‐related vascular dysfunction and reveal new therapeutic targets.

In addition to mechanistic studies, validating the clinical utility of impedance‐based leukocyte profiling in larger, multi‐center cohorts is essential. While our findings demonstrate its potential for vascular risk stratification in diabetes and CVD (Figure 6), further studies across diverse populations will be necessary to establish its robustness and predictive power. Expanding investigations across different ethnic groups, age ranges, and disease severities in larger cohorts could help refine risk thresholds and assess feasibility for future clinical translation, ensuring eventual accessibility and reliability in routine healthcare settings.

Furthermore, integrating impedance‐based cell profiling with multi‐omics approaches such as proteomics and metabolomics could significantly enhance biomarker discovery for vascular complications in diabetes and provide a more comprehensive understanding of immune dysfunction in metabolic diseases. Such an integrative approach may facilitate personalized diagnostics, improving early intervention strategies for high‐risk individuals and advancing precision medicine for diabetes and CVD management.

## 4 Experimental Section

### DFF Device Operation—Human Whole Blood Sorting

Whole blood was diluted (1:10 v/v) in 0.1% bovine serum albumin (BSA, Miltenyi Biotec) in phosphate‐buffered saline (PBS, Lonza) and perfused into the microfluidics device along with sheath fluid (0.1% BSA) using syringe pumps (Harvard Apparatus) at flow rates of 140 and 1400 µL min^−1^, respectively. Sample collections were performed at outlet 2 after 1 min of flow stabilization. For monocyte and lymphocyte fractionation, PBMCs were first isolated using Ficoll–Paque (Cytiva) according to the manufacturer's protocol. The PBMCs were resuspended in 0.1% BSA and fractionated at sample and sheath flow rates of 130 µL min^−1^ (sample) and 1300 µL min^−1^ (sheath). Monocytes were collected from outlet 2, while lymphocytes were collected from outlet 3 after 1 min of flow stabilization.

### DFF Device Operation—Mouse Whole Blood Sorting

200 µL of whole blood was diluted in 0.1% BSA and perfused through the DFF device along with sheath buffer at flow rates of 115 and 1150 µL min^−1^, respectively. Mouse neutrophils were collected at outlet 2 after 1 min of flow stabilization.

### Multi‐Frequency Electro‐Mechano‐Phenotyping Principle

The developed impedance deformability cytometry design incorporated two pairs of electrodes and a unique hydrodynamic cell stretching feature, enabling two‐point recordings to extract cellular properties at their native and deformed states.^[^ ^26^ ^]^ The developed impedance cytometry utilized multi‐frequency (0.3, 1.72, and 12 MHz) electrical signals, facilitating a thorough screening of cellular biophysical properties across different cellular components. Briefly, the impedance response from low frequency (0.1–1 MHz) to moderate frequency (1–10 MHz) to high frequency (> 10MHz) was dominated by cellular components from the whole cell level to the cell membrane to cytoplasmic components (cytoplasmic and nucleus).^[^ ^65^ ^]^ Specifically, cell size was characterized by the impedance magnitude at low frequency in undeformed state (|Z|_0.3MHz pre‐deform_), membrane opacity was determined by the ratio of moderate frequency to low frequency in undeformed state (|Z| _1.72 MHz pre‐deform_/|Z| _0.3MHz pre‐deform_), nucleus opacity was calculated by the ratio of high frequency to low frequency in undeformed state (|Z| _12 MHz pre‐deform_/|Z| _0.3MHz pre‐deform_), and deformability index was detected by the ratio of magnitude attenuation after deformation at low frequency (|Z| _0.3 MHz pre‐deform_/|Z|_0.3MHz post‐deform_) (Figure 1a). Therefore, through co‐excitation of electrical signal across these ranges, cellular key components such as cell size, deformability, membrane opacity and nucleus opacity were characterized.

### Impedance Cytometry Operation—Human Leukocytes Measurement

Human leukocytes were suspended in 0.75 wt.% poly(ethylene oxide) (PEO) (Sigma–Aldrich, MW 600000) in PBS at a concentration of 1 million cells mL^−1^, with 10 µm polystyrene beads added as a standard for cell size. The sample and sheath buffer (0.75 wt.% PEO in PBS) were perfused through the microfluidics devices at flow rates of 5 and 15 µL min^−1^, respectively, and subjected to 3 min of data acquisition, with measurements taken at a sampling rate of 115 kS s^−1^ (LabVIEW). Flow stabilization was ensured with a minimum idle time of 3 min between samples. During measurement, the microfluidics device was connected to an in‐house designed printed circuit board where a lock‐in amplifier (HF2LI, Zurich Instruments) would excite the samples with 1V at three different frequencies. A trans‐impedance amplifier (HF2TA, Zurich Instruments) converted the collected current signals to impedance signals and fed them back to the lock‐in amplifier to determine the differential signal amplitude and phase of both electrodes.

### Impedance Cytometry Operation—Mouse Neutrophils Measurement

Sorted mouse neutrophils were suspended in 1.25 wt.% PEO in 1× PBS at a concentration of 1 million cells mL^−1^, with 7 µm polystyrene beads added as an internal standard. The sample and sheath buffer (1.25 wt.% PEO in PBS) were perfused through the microfluidics devices at flow rates of 2 and 10 µL min^−1^, respectively, with 5 min of data acquisition and a sampling rate of 50 kS s^−1^. Flow stabilization was ensured with a minimum idle time of 5 min between samples. The same measurement parameters (frequencies, voltage) used for human leukocyte phenotyping were applied.

### Cell Treatment—Neutrophil Treatment

Isolated neutrophils (1 million) were washed and resuspended in Roswell Park Memorial Institute (RPMI) medium (Gibco) and treated with glucose (30 mm) to mimic a high glucose environment, and withTNF‐α (0.1 ng mL^−1^, Peprotech) to induce inflammation. Untreated cells in culture medium served as the control. All treatment conditions and control neutrophils were incubated at 37 °C with 5% CO_2_ for 120 min.

### Cell Treatment—NPAs Formation

To study the biophysical properties of NPAs and mimic pro‐thrombotic conditions, blood was collected from healthy donors via venipuncture using BD Vacutainer tubes with sodium citrate. The collected whole blood was gently shaken to ensure homogeneous mixing of plasma and blood cells, then aliquoted. The aliquots were treated with TRAP‐6 (20 µm, Sigma–Aldrich) for 20 min at room temperature, while one aliquot remained untreated as a control.

### UMAP Analysis

Five hundred cells were randomly selected from each sample, and nine biophysical features of each cell were imported into Python (version 3.9.7). Standard z‐scores were used to normalize each biophysical feature before dimensional reduction. UMAP analysis was performed with a number of neighbors set to 20 and a minimum distance of 0.1, producing a two‐parameter (UMAP1 and UMAP2) representative plot.

### Statistical Analysis

All statistical analyses were performed using GraphPad Prism 10.0.2. Data were analyzed with Mann–Whitney test or Student's *t*‐test and presented as mean ± SEM. Pearson correlation was used for correlation analysis. Statistical significance was annotated with one of the following. *ns*: not significant, ^*^ *P* < 0.05, ^**^ *P* < 0.01 ^***^ *P* < 0.001, ^****^ *P* < 0.0001.

Details of the microfluidic chip fabrication, animal housing and treatment, staining protocols, and RNA sequencing are provided in the Supporting Information.

### Study Approval

Written informed consent was obtained for all subjects during recruitment. All protocols were approved by the institutional review board of Nanyang Technological University (IRB‐2019‐03‐011, IRB‐2021‐01‐037), Tan Tock Seng Hospital (DSRB 2018/00880, DSRB 2020/01324) in compliance with the Human Biomedical Research Act (Ministry of Health, Singapore).

Experimental protocols of animal studies were reviewed and approved by Nanyang Technological University Institutional Animal Care and Use Committee (Protocol A19051).

## Conflict of Interest

The authors declare no conflict of interest.

## Author Contributions

L.H. did conceptualization, methodology, investigation, data analysis and writing. H.M.T. did conceptualization, methodology, investigation and data analysis. F.C. did investigation, methodology and data analysis. H.S.C. did data analysis. L.D.W. did investigation. Q.N., A.T., and L.G. did investigation. A.C. and N.S.T. did supervision. K.H.H.L. did supervision and financial support. R.D. and S.L.W. did supervision, financial support, and review. H.W.H. did conceptualization, methodology, supervision, financial support, writing, and review

## Supporting information



Supporting Information

## Data Availability

The data that support the findings of this study are available from the corresponding author upon reasonable request.
