# Supplementary material for: Label‐Free Leukocyte Biophysical Profiling Using Impedance‐Deformability Cytometry for Rapid Cardiovascular Risk Stratification
Source: Adv Sci (Weinh). 2025 Nov 28;13(9):e16021. doi: 10.1002/advs.202516021 (PMC12904061; doi:10.1002/advs.202516021)
Supplement: Supplementary file 1 — Supporting Information [file ADVS-13-e16021-s001.docx]

**Label-free Leukocyte Biophysical Profiling Using Impedance-deformability Cytometry for Rapid Cardiovascular Risk Stratification**

Linwei He^1^, Hui Min Tay^1^, Feng Chen^2^, Hong Sheng Cheng^2^, Liang De Wang^2^, Qiqi Nam^2^, Arunachalam Thannirmalai^1^, Lingyan Gong^1^, Aram J. Chung^3^, Nguan Soon Tan^2,4^, King Ho Holden Li^1^, Rinkoo Dalan^5^, Siu Ling Wong^2,6^, Han Wei Hou^1,2*^

^1^School of Mechanical and Aerospace Engineering, Nanyang Technological University, Singapore

^2^Lee Kong Chian School of Medicine, Nanyang Technological University, Singapore

^3^School of Biomedical Engineering, Korea University, Seoul, Republic of Korea

^4^School of Biological Sciences, Nanyang Technological University, Singapore

^5^Endocrinology Department, Tan Tock Seng Hospital, Singapore

^6^Tan Tock Seng Hospital, National Healthcare Group, Singapore

**Corresponding authors:* [*hwhou@ntu.edu.sg*](mailto:hwhou@ntu.edu.sg) *(Han Wei Hou)*

# Contents

**Supporting Experimental Section**

**Supporting Figures 1-18**

Fig. S1 | Association of impedance deformability index with HL-60 cells rigidity

Fig. S2 | Efficacy of DFF

Fig. S3 | Representative impedance scatter plots of different biophysical parameters measured for DFF-sorted single leukocytes

Fig. S4 | Representative impedance scatter plots of neutrophil biophysical properties against cell size after glucose and TNF-α treatments

Fig. S5 | Normalized mean cell size, membrane opacity, and nucleus opacity for untreated neutrophils, and neutrophils treated with glucose (Glu) and TNF-α

Fig. S6 | Impedance-based gating strategy for identifying neutrophils from residual RBCs after DFF

Fig. S7 | Analysis of leukocyte-platelet aggregates (LPAs) biophysical property alterations

Fig. S8 | Impedance scatter plot for diluted blood before and after DFF sorting

Fig. S9 | Measurements of mouse biological parameters

Fig. S10 | Impedance-based biophysical properties changed of mouse neutrophils from week 8 to week 12

Fig. S11 | Nucleus opacity and deformability index for neutrophils isolated from wild-type controls (*WT Veh*), mice (*WT STZ*), and ApoE knockout model with diabetes (*ApoE KO STZ*) at week 8

Fig. S12 | UMAP parameters and magnitude mapping for mouse neutrophils in diabetes atherosclerotic mouse model (*ApoE KO STZ* mice)

Fig. S13 | Fluorescence images of DNA and Ly6G^+^ cells in mouse liver

Fig. S14 | Fluorescence images of DNA and ICAM-1 positive area in mouse liver

Fig. S15 | Changes in lymphocytes associated with disease severity

Fig. S16 | Changes in monocytes associated with disease severity

Fig. S17 | UMAP parameters and magnitude mapping for neutrophils isolated from Ctrl, Pre-DM, DM and DM-CVD subjects

Fig. S18 | PCA score association with clinical parameters

**Supporting Tables 1-2**

Table S1 | Donor recruitment criteria

Table S2 | Clinical characteristics of participants

# Supporting Experimental Section

**Size-based cell fractionation principle:**

Our group has previously reported a Dean Flow Fractionation (DFF) device (fabrication details in SI) ^[1]^ that leverages cell size-sensitive inertial forces ($F_{l}$) and drag force ($F_{d}$) to laterally separate the cells based on their size. As shown in Fig. 1a and b, cell focusing is dominated by inertial forces at high flow rates, allowing larger cells to be concentrated closer to the inner wall of the spiral channel while smaller cells are positioned further away. Using this DFF device, neutrophils (~10 µm) were effectively separated from RBCs (~6 µm), while monocytes (~12 µm) and lymphocytes (~7 µm) were successfully fractionated from peripheral blood mononuclear cells (PBMCs).

**Device fabrication:**

*DFF device fabrication:* The two-inlet, four-outlet Dean Flow Fractionation (DFF) spiral device was created using standard soft lithography techniques with poly(dimethylsiloxane) (PDMS, Sylgard 184, Dow Corning), as previously reported by our group ^[1]^. PDMS base was mixed with a curing agent at a 10:1 (w/w) ratio and poured over a silicon wafer patterned with a channel design (105 µm height). The mixture was cured at 75°C for 45 min before being peeled off. Inlet and outlet holes (1.5 mm) were punched with a biopsy puncher, and the PDMS slab was cleaned with isopropyl alcohol (IPA) and subjected to plasma treatment using an air plasma machine. The PDMS slab was then bonded to a 1 mm thick glass substrate (70 mm × 50 mm) and baked for an additional hour.

*Impedance cytometry fabrication:* The impedance cytometry device featured two inlets and one outlet, designed with square microchannels (45 µm width and height) for human leukocytes and rectangular microchannels (45 µm width and 25 µm height) for mouse leukocytes. Devices were fabricated using PDMS via standard soft lithography and bonded to a glass substrate with patterned gold electrodes ^[2, 3]^. Gold electrodes were created by sputtering Au (200 nm) and Cr (20 nm) onto JGS2 quartz slides. The printed circuit board (PCB) was made from an FR-4 Tg 130-140 circuit board with gold immersion. PDMS was fabricated as previously described and plasma-treated for 2 min before being aligned and bonded to the electrodes, followed by another hour of baking at 75 °C.

**Magnetic active cell sorting:**

Whole blood from heath donors was added with 1 mM EDTA (Life Technologies) and neutrophils were isolated using EasySep™ direct human neutrophil isolation kit (Stemcell Technologies) according to manufacturer’s protocol. The collected cells were suspended in 0.1% BSA in PBS.

**Animal housing and treatment:**

Male *db/m+* and *db/db* mice (4–5 weeks old), WT (C57BL/6J) and *Apoe-/-* mice (7 weeks old) were purchased from The Jackson Laboratory (Maine, United States) and housed under specific pathogen-free (SPF) conditions with free access to chow diet and water. After one week of acclimatization, body weight and free-fed blood glucose levels were measured weekly for *db/m+* and *db/db* mice, with 200 µL of blood collected biweekly from the retro-orbital plexus using a glass capillary starting at week 8 for microfluidic assessments. For WT and *Apoe-/-* mice, after one week of acclimatization, intraperitoneal injections of either vehicle (Veh, 0.1 M sodium citrate buffer, pH 4) or streptozotocin (*STZ*, 50 mg/kg per day) were administered for five consecutive days. Body weight and free-fed blood glucose levels were measured at 3 and 5 weeks post-*STZ* induction. Only mice with free-fed blood glucose levels exceeding 16 mmol/L were included in the study. Approximately 200 µL of blood was collected from the retro-orbital plexus staring at 8 weeks post-*STZ* induction for microfluidic assessments. Mice were euthanized at 12 weeks of age (*db/db* and db/m+) or 12 weeks post-*STZ* induction (WT and *Apoe-/-*) for liver sample collection.

**Cardiovascular disease (CVD):**

CVD was defined as a documented history of cardiovascular events, including previous acute myocardial infarction, percutaneous coronary intervention (PCI), or hospitalization for heart failure, confirmed through medical records and clinician diagnosis.

**Staining:**

*F-actin staining:* Treated cells were fixed with 4% paraformaldehyde (PFA) at room temperature for 15 min and permeabilized with 0.1% Triton-X (Sigma-Aldrich) in PBS for 5 min. After permeabilization, cells were washed and stained with Hoechst 33342 (1 μg/mL, Life Technologies) and AlexaFluor 568 phalloidin (0.17 μM, Life Technologies) for 30 min. The stained cells were washed with PBS before flow cytometry analysis (Fortessa X-20, BD Biosciences). Unstained samples served as negative controls.

*NPA staining:* TRAP-6 treated cells and corresponding control samples collected from DFF were stained with Hoechst 33342 (1 μg/mL), FITC-conjugated anti-human CD41a (1:20, eBioscience), and APC-conjugated anti-human CD66b (1:20, eBioscience) at 4 °C for 30 min to identify nuclei, platelets, and neutrophils, respectively. The stained cells were washed with 0.1% BSA and spotted on a glass slide for imaging (Nikon Eclipse Ti) and flow cytometry analysis. Phase contrast and fluorescence images were captured at 100× magnification using MetaMorph software (Molecular Devices) and processed using ImageJ.

*Mouse leukocytes immunofluorescence staining:* Diluted mouse blood (control) and sorted mouse blood from DFF outlet 2 were co-stained with APC-conjugated anti-mouse Ly6G (1:20, Biolegend) and FITC-conjugated anti-mouse CD45 (1:20, Biolegend) at 4°C for 30 min to quantify neutrophils and leukocytes, respectively. The stained cells were washed with 0.1% BSA and used for flow cytometry analysis. Isotype controls were selected according to the manufacturer’s recommendations.

*Mouse organ immunofluorescence staining:* Liver samples were preserved in optimal cutting temperature (OCT) compound for visualization and quantification of neutrophil infiltration, neutrophil-platelet aggregates, and endothelium activation. Briefly, the liver samples were cut on a cryostat into 10 µm sections and fixed in zinc fixative overnight at room temperature. Sections were then permeabilized with 0.1% sodium citrate (S1804, Sigma-Aldrich) in PBS (BUF-2041-10X1L, 1st Base) containing 0.1% Triton X-100 (T8787, Sigma-Aldrich) and blocked with 3% BSA (A7906, Sigma-Aldrich) for 1 h. Primary antibody (rat anti-mouse Ly-6G, BD Bioscience, 551459, 1:500; rabbit anti-mouse CD42b Polyclonal Antibody, ProteinTech, 12860-1-AP, 1:500; rabbit anti-mouse ICAM-1/CD54 Polyclonal antibody, ProteinTech, 10020-1-AP, 1:200) diluted in 0.3% BSA with 0.05% Tween-20 (P9416, Sigma-Aldrich) were incubated with liver sections overnight at 4°C. Secondary antibodies (Alexa Fluor 555 goat anti-rat IgG (H+L) secondary antibody, Invitrogen, A21434, 1:1500; Alexa Fluor 488 goat anti-rabbit IgG (H+L) cross-adsorbed secondary antibody, Invitrogen, A11008, 1:1500) were applied on the following day, where appropriate. The sections were then counter-stained with Hoechst 33342 (H3570, Sigma-Aldrich), and mounted with fluorescence mounting medium (17985-10, Electron Microscopy Sciences). Images were then acquired on inverted fluorescence microscope (Axio observer 7, Carl Zeiss) using the Plan Apochromat 63x/1.4 oil lens for quantification. Number of neutrophils infiltrated into the liver, number of neutrophil-platelet aggregates, and ICAM-1 high area (%) were quantified from 11 high-power fields.

*Mouse organ histology staining:* Liver samples were embedded in 4% PFA and embedded in paraffin. To investigate the pathological features of liver, hematoxylin and eosin (H&E) staining was performed on formalin-fixed paraffin-embedded (FFPE) samples of 12-week-old *db/m+* and *db/db* mice. Five µm liver sections were cut on microtome (RM2245, Leica) and deparaffinised followed by hematoxylin (Surgipath Hematoxylin Gill II, 38016SS4C, Leica Biosystems) and eosin (HT110116, Sigma-Aldrich) staining. Sections were then dehydrated by xylene (3803600, Leica Biosystems) and mounted with DPX mountant (06522, Sigma-Aldrich). Images were acquired on slide scanner using Plan Apochromat 20x/0.8 (Axio Scan.Z1, Carl Zeiss).

*Human leukocytes immunofluorescence staining:* Leukocytes sorted by DFF were stained with APC-conjugated anti-human CD66b (1:20, eBioscience), PE- conjugated anti-human CD14 (1:20, eBioscience), and FITC-conjugated anti-human CD3 (1:20, eBioscience) and CD19 (1:20, eBioscience) for 30 min at 4°C to stain neutrophils, monocytes, and lymphocytes, respectively. All stained cells were washed once with 1X PBS and analyzed using a BD LSR flow cytometer.

**Bulk RNA sequencing:**

RNA was extracted from MACS-isolated neutrophils using RNA extraction kit (Qiagen). Total RNA was dissolved in RNAase-free water and sent for bulk RNA sequencing (Novogene, Singapore). Differential gene expression was performed using DESeq2 ^[4]^. Genes with a false discovery rate (FDR) < 0.05 and log_2_ fold change > ± 1 were considered differentially expressed genes (DEGs). Functional enrichment analysis was carried out using ViSEAGO and Gene Set Enrichment Analysis (GSEA) ^[5]^ while disease ontology enrichment analysis was performed using DOSE ^[6]^.

# Supporting Figures

Impedance deformability association with cytoskeletal integrity

To test if impedance-quantified cell deformation correlates with cytoskeletal integrity in neutrophils (~10 to 12 µm), the human leukemia cell line (HL-60) was used. Cells were fixed with PFA, a commonly used cell cross-linking agent, to represent stiffer cells, and treated with latrunculin B (Lat B) ^[7]^, an actin inhibitor, and blebbistatin (BB) ^[8]^, a myosin II inhibitor, to mimic cells with reduced rigidity. A reduction in electrical deformability was observed for PFA-treated cells, while a significant increment of deformability was noted for Lat B and BB treatments (**Fig. S1a**). Flow cytometry analysis confirmed that Lat B treatment significantly reduced F-actin intensity, indicating effective disruption of cytoskeletal integrity (Fig. S1b). Repetitive experiment shows that the deformability index increased with higher sheath pinching ratios for all samples, and distinct alterations in the deformability index were observed regardless of the operational parameters. To avoid excessive cell deformation that might damage cell surface integrity, a sheath-to-sample ratio of 3 was selected for subsequent experiments. Under this condition, the mean deformability indices were 1.10 ± 0.033, 1.05 ± 0.006, 1.13 ± 0.028, and 1.11 ± 0.028 for control, PFA-, Lat B-, and BB-treated HL-60 cells (n=3), respectively, confirming a strong association between the impedance deformability index and cell rigidity (Fig. S1c).


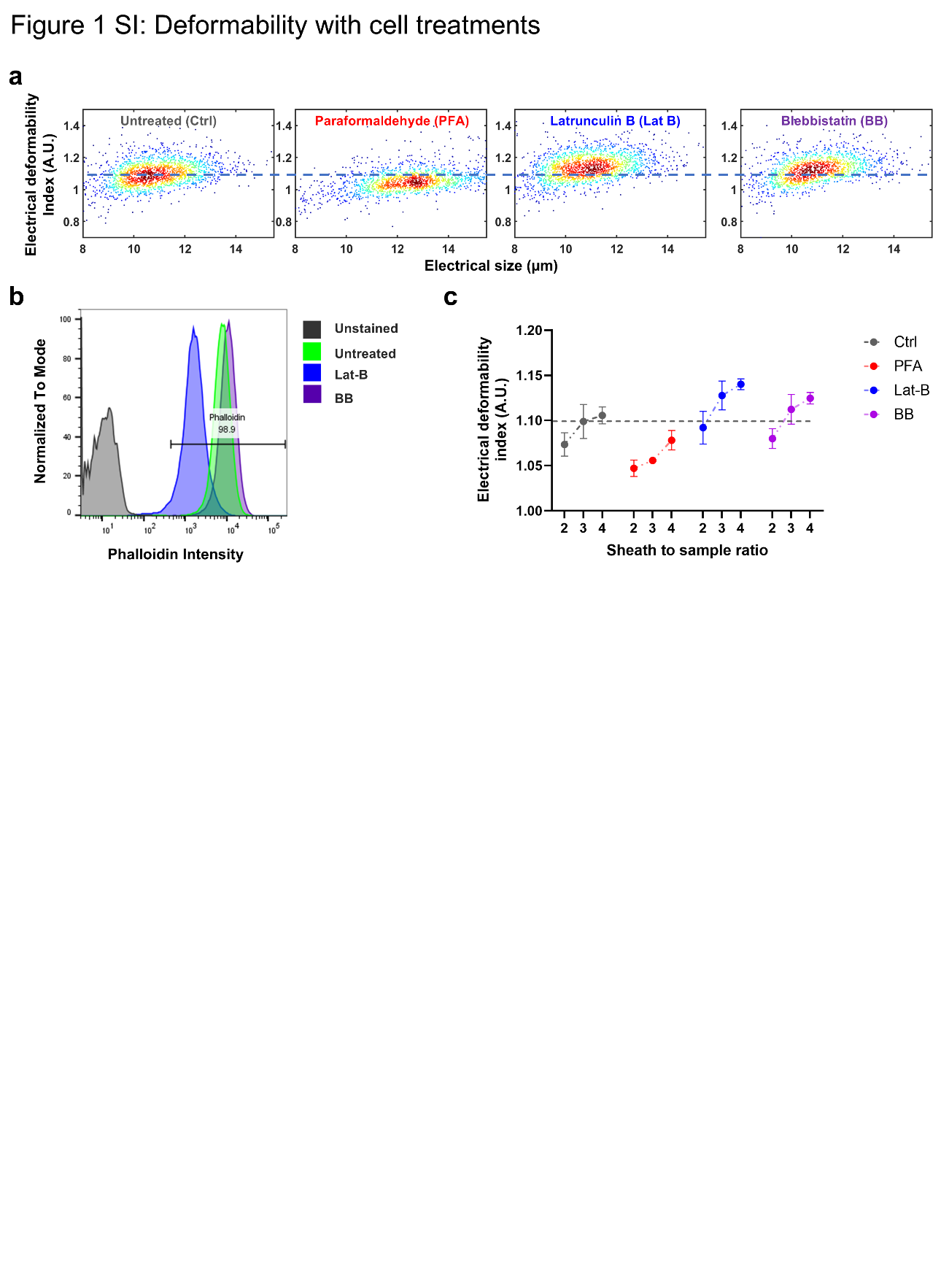


**Fig. S1: Association of impedance deformability index with HL-60 cells rigidity. a)** Scatter plots of the deformability index and electrical size for HL-60 cells under different treatment conditions (dotted line represented the mean value of control). **b)** F-actin staining intensity plot for unstained, control, Lat B-treated, and BB-treated HL-60 cells. **c)** Electrical deformability index for HL-60 with different treatment conditions measured at various flow rate (n=3).


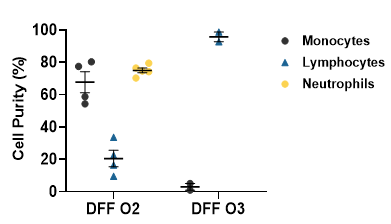


**Fig. S2:** Efficacy of DFF in isolating neutrophils from whole blood and monocytes and lymphocytes from peripheral blood mononuclear cells (PBMCs).


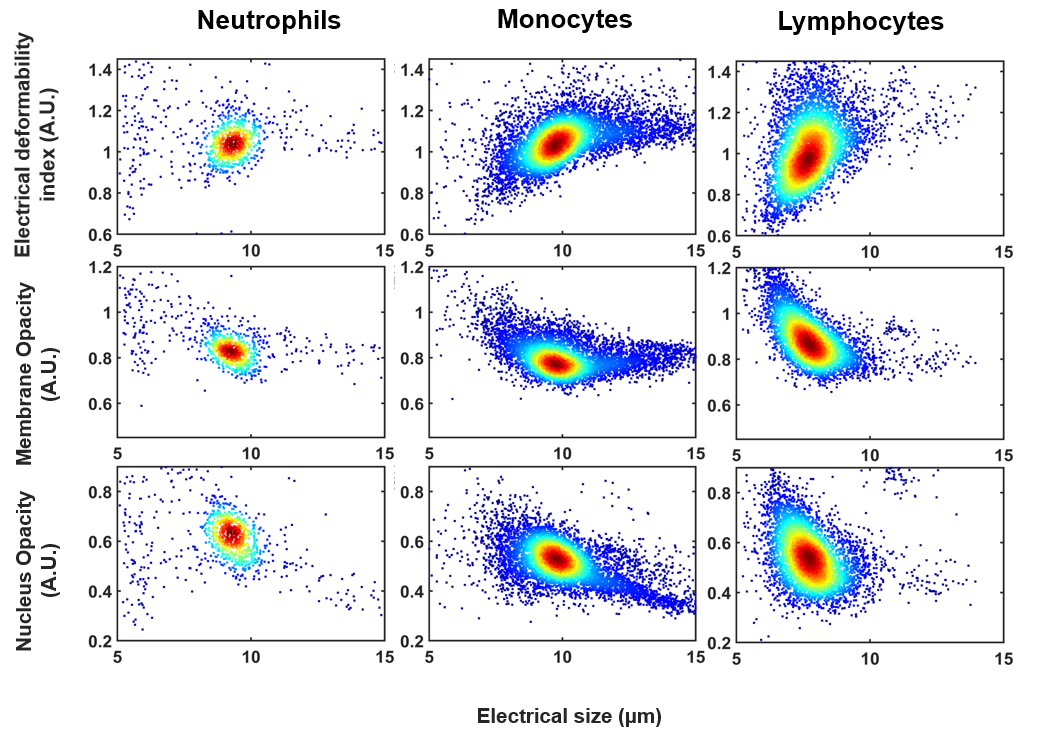


**Fig. S3**: Representative impedance scatter plots of different biophysical parameters measured for DFF-sorted single leukocytes (neutrophils, monocytes, and lymphocytes).


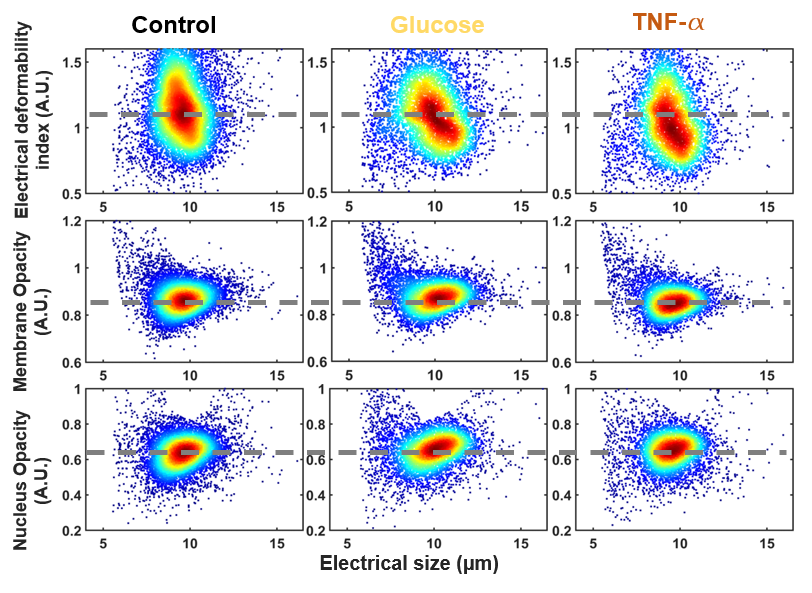


**Fig. S4:** Representative impedance scatter plots of neutrophil biophysical properties (deformability, membrane opacity, and nucleus opacity) against cell size after glucose and TNF-α treatments. (The dotted line represents the mean of the control group.)


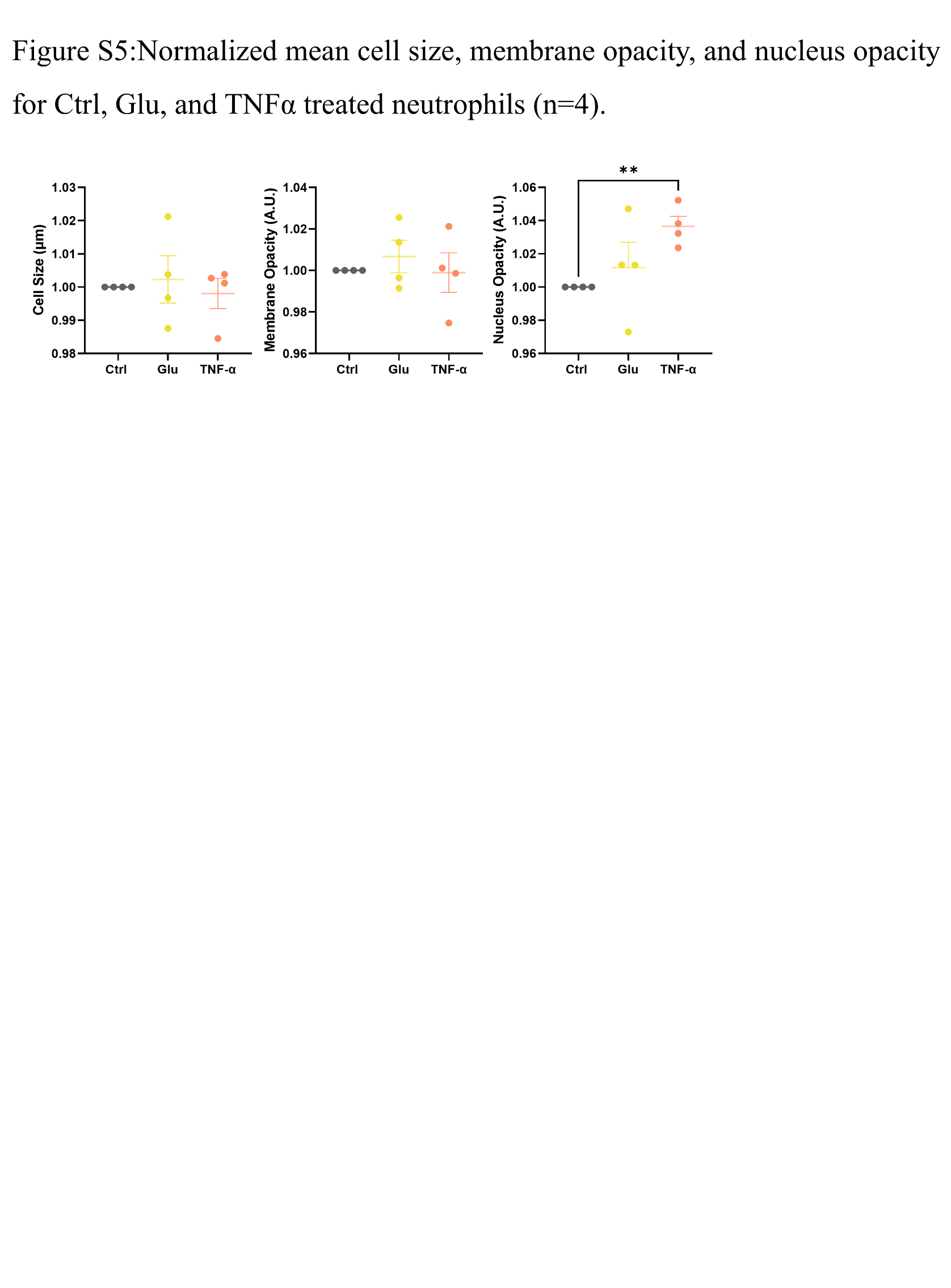


**Fig. S5:** Normalized mean cell size, membrane opacity, and nucleus opacity for untreated neutrophils as control (Ctrl), and neutrophils treated with glucose (Glu) and TNF-α (n = 4).

Impedance gating

DFF sorting depleted bulk of the RBCs from whole blood and the DFF-sorted O2 cells showed 2 distinct populations of residual RBCs and enriched neutrophils in the impedance scatter plot, facilitating easy neutrophil gating based on their cell size and deformability (Fig. S6).


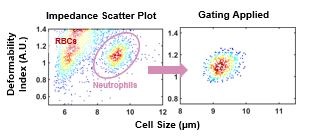


**Fig. S6:** Impedance-based gating strategy for identifying neutrophils from residual RBCs after DFF.

Leukocytes biophysical properties alteration after TRAP-6 treatment

Whole blood samples were treated with TRAP-6 and were fractionated using immunomagnetic negative selection (RBC depletion reagent, STEMCELL) without pre-addition of anticoangulant (EDTA). Leukocyte-platelet aggregates (LPAs) were observed for both control and TRAP-6 treated samples with higher LPAs abundance after TRAP-6 treatment (Fig. S7a). 500 single cell readouts were randomly selected from each sample and violin plots were ploted as shown in Fig. S7b,where TRAP-6 treated leukocytes showed changes in size, membrane opacity and deformability index while no significant change was observed for nucleus opacity.


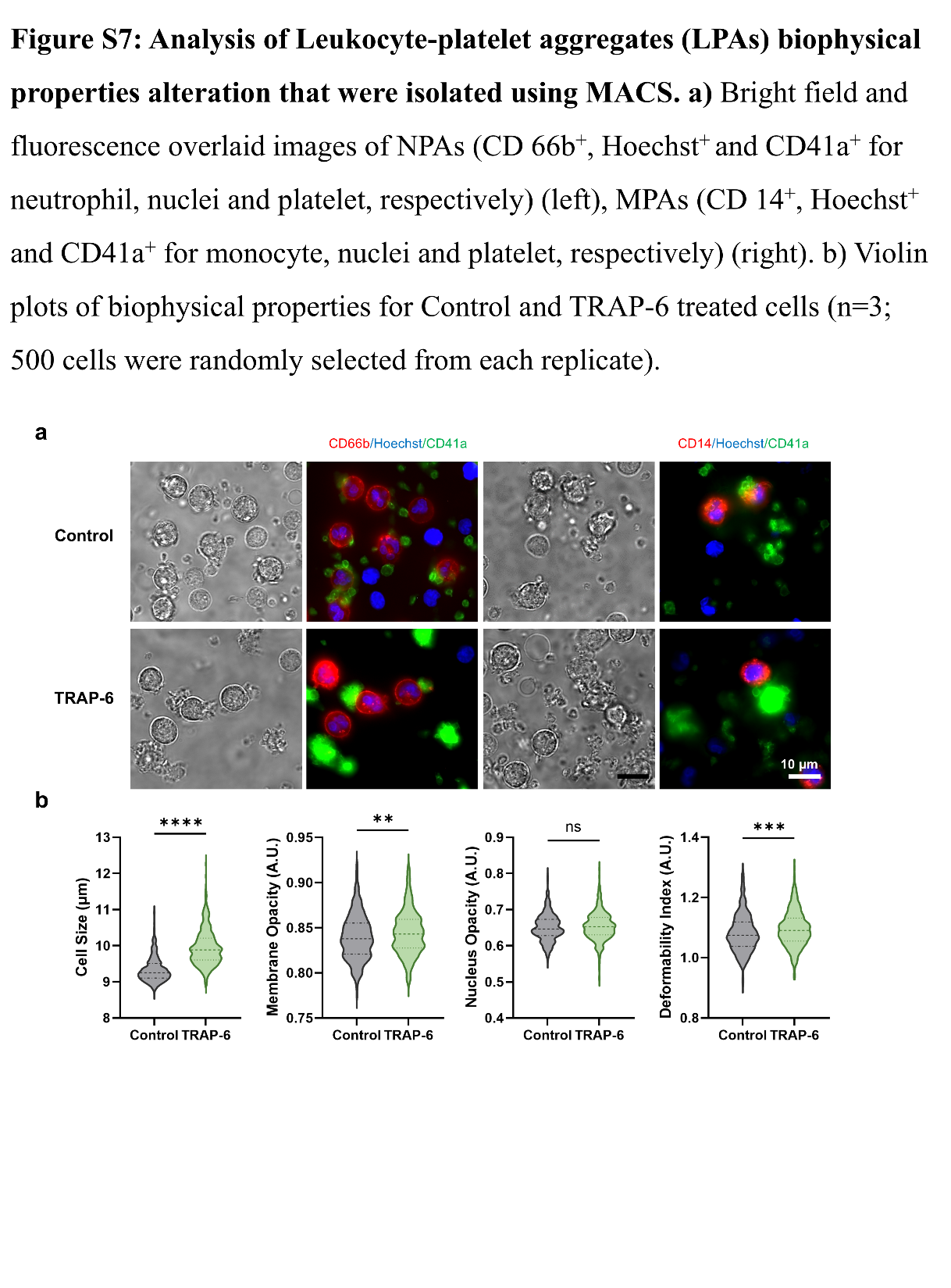


**Fig. S7: Analysis of leukocyte-platelet aggregates (LPAs) biophysical property alterations. a)** Bright field and fluorescence overlay images of NPAs (CD 66b^+^, Hoechst^+^ and CD41a^+^ for neutrophil, nuclei and platelet, respectively) (left), MPAs (CD 14^+^, Hoechst^+^ and CD41a^+^ for monocyte, nuclei and platelet, respectively) (right). **b)** Violin plots of biophysical properties for control and TRAP-6 treated cells (n=3; 500 cells were randomly selected from each replicate).


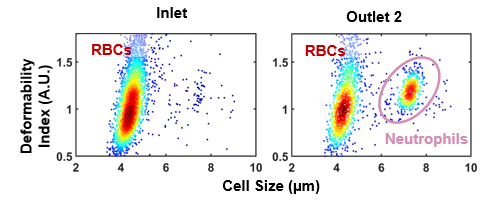


**Fig. S8:** Impedance scatter plot for diluted blood before (left) and after (right) DFF sorting. The pink gate was used for classifying neutrophils.

Mouse biological parameters in type 2 diabetes (*db/db*) mouse model

Both control (*db/m+)* and diabetic (*db/db)* mice increased in body weight from week 6 to week 12, with *db/db* mouse gaining weight significantly faster (Fig. S9a, left). Also, the *db/db* mice showed significantly higher non-fasting glucose level than *db/m+*, confirming diabetic development in the *db/db* mice (Fig. S9a, right). At week 12, *db/db* mice exhibited a noticeable increase in liver weight compared to *db/m+* controls (Fig. S9b, n = 7), suggesting potential hepatic changes such as steatosis observed in obese and diabetic conditions (also reflected in Fig. 3f).


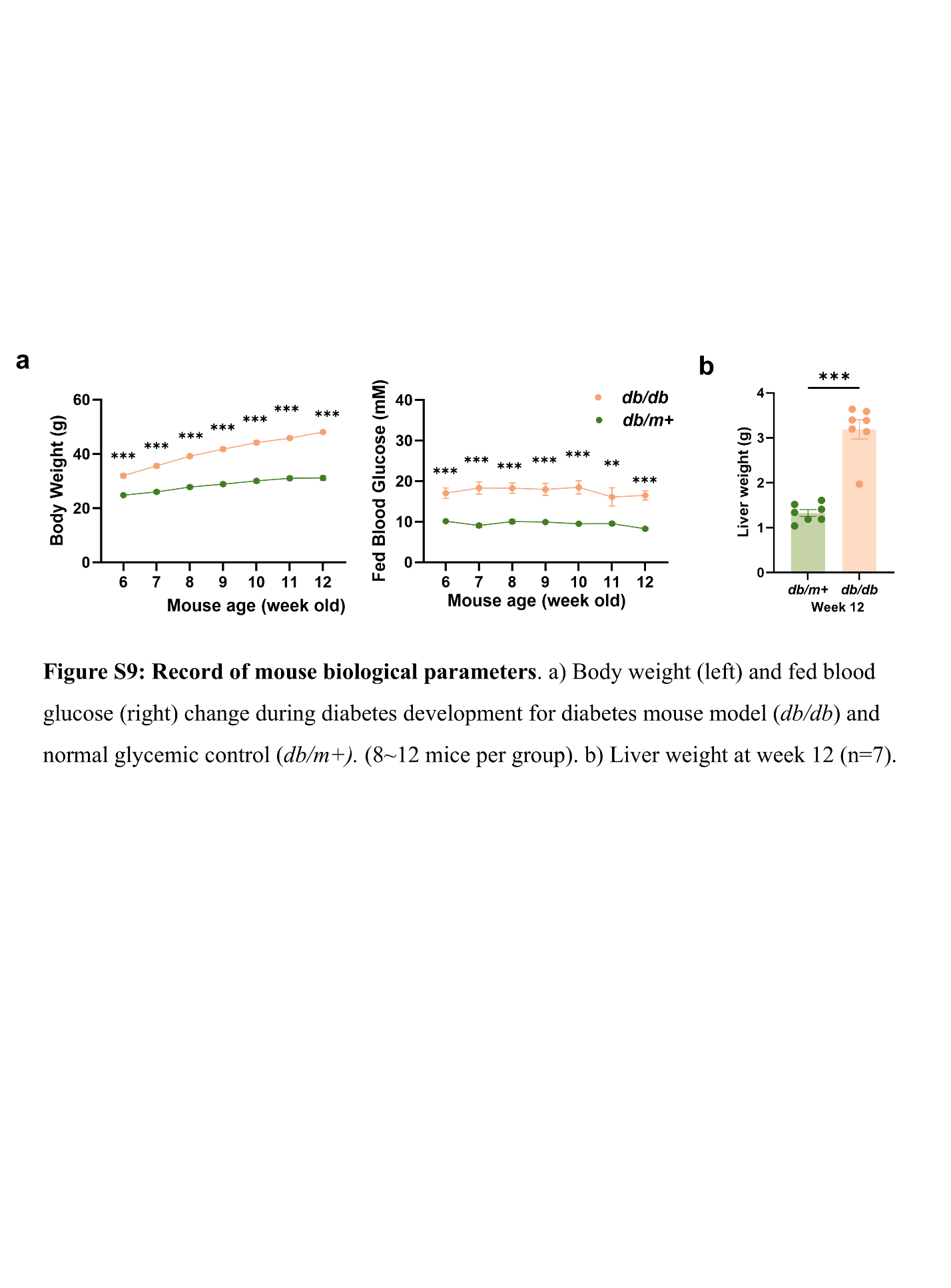


**Fig. S9: Measurements of mouse biological parameters. a)** Body weight (left) and fed blood glucose (right) change during diabetes development for diabetes mouse model (*db/db*) and normal glycemic control (*db/m+).* (8~12 mice per group). **b)** Liver weight at week 12 (n=7). **P < 0.01, ***P < 0.001 based on Mann Whitney test (a) or student’s t-test (b).


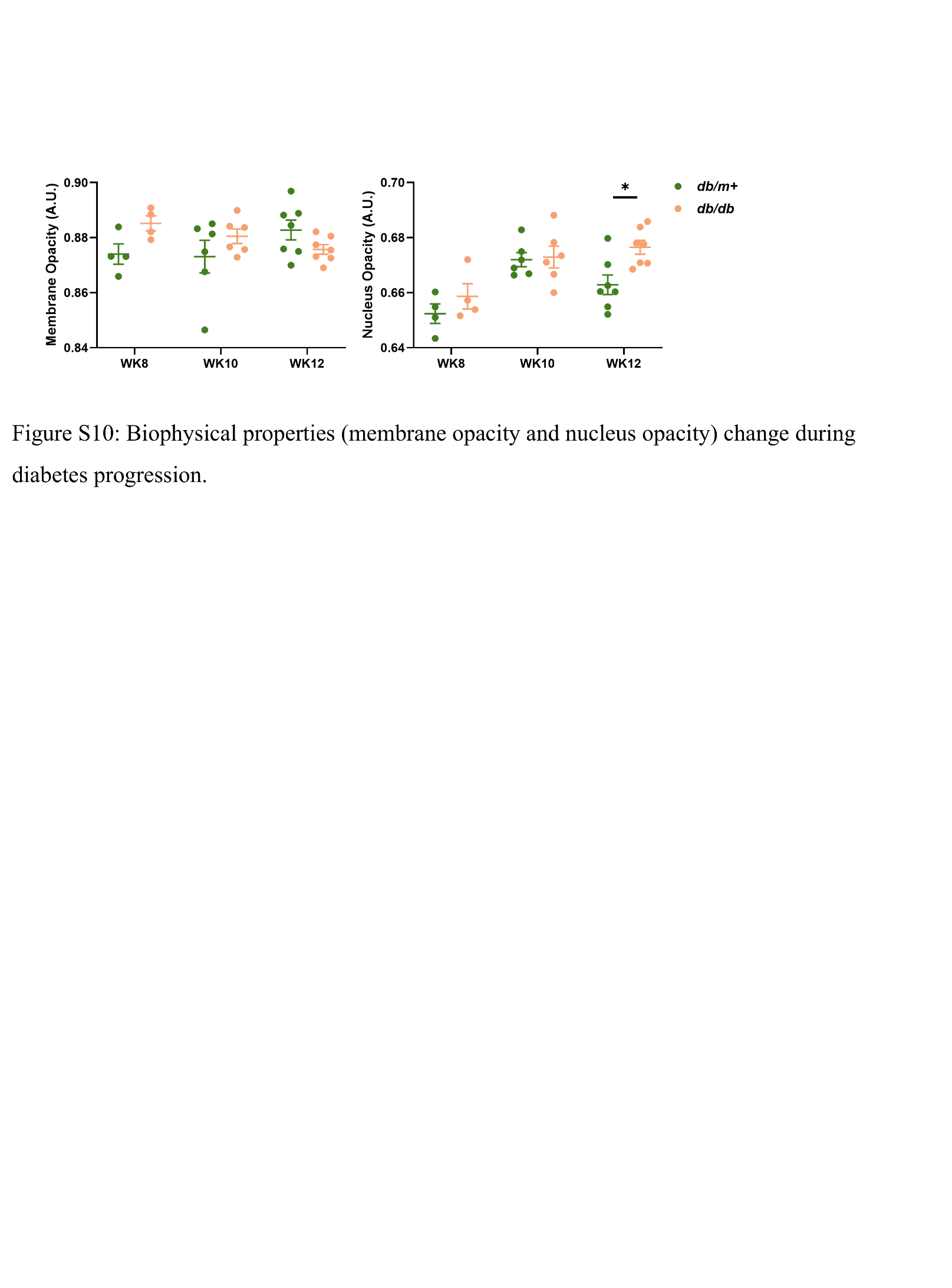


**Fig. S10:** Impedance-based biophysical properties (membrane opacity and nucleus opacity) changed of mouse neutrophils from week 8 to week 12.


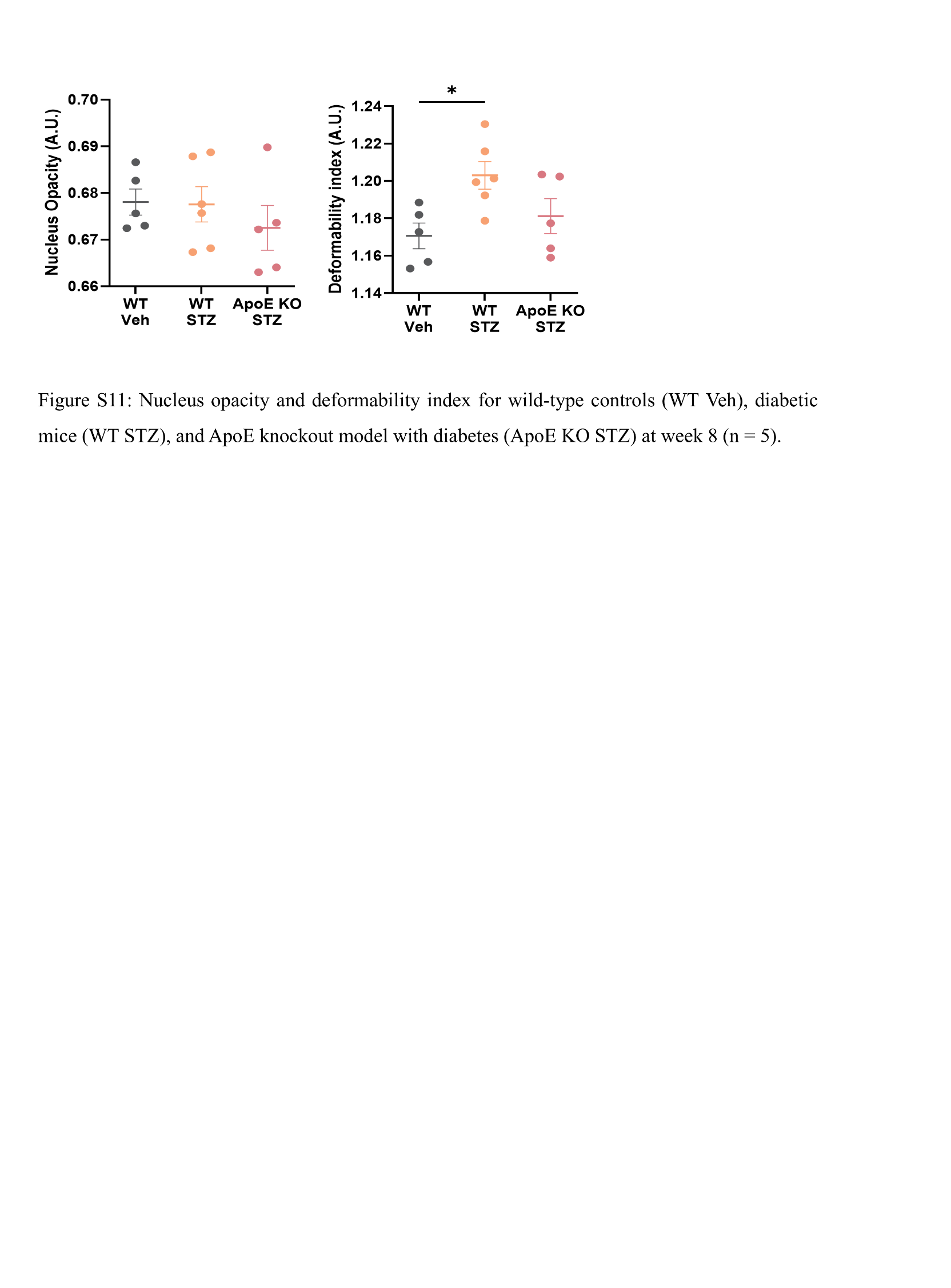


**Fig. S11:** Nucleus opacity and deformability index for neutrophils isolated from wild-type controls (*WT Veh*), mice (*WT STZ*), and ApoE knockout model with diabetes (*ApoE KO STZ*) at week 8 (n = 5/group).


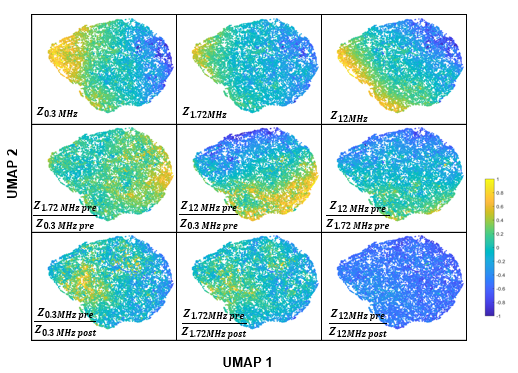


**Fig. S12:** UMAP parameters and magnitude mapping for mouse neutrophils in diabetes atherosclerotic mouse model (*ApoE KO STZ* mice)


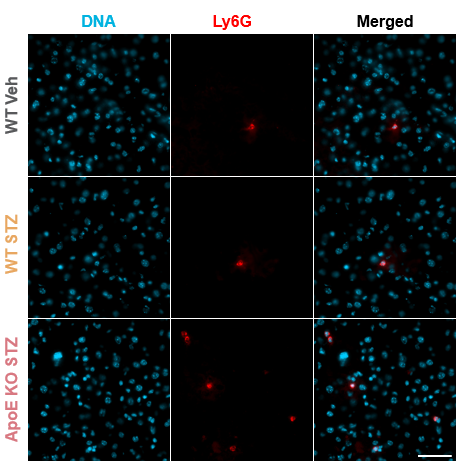


**Fig. S13:** Fluorescence images of DNA and Ly6G^+^ cells in mouse liver (Scale bar: 50µm).


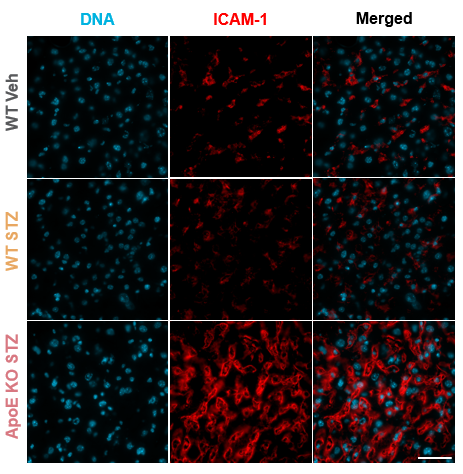


**Fig. S14:** Fluorescence images of DNA and ICAM-1 positive area in mouse liver (Scale bar: 50 µm).

Alteration of biophysical properties in DM-CVD lymphocytes

The biophysical properties of lymphocytes are shown in **Fig. S15**a. As opposed to DM-CVD neutrophils, DM-CVD lymphocytes exhibited more significant changes in nucleus opacity and cell deformability. This led to slight separation of DM lymphocytes and DM-CVD lymphocytes in UMAP analysis (Fig. S15b).


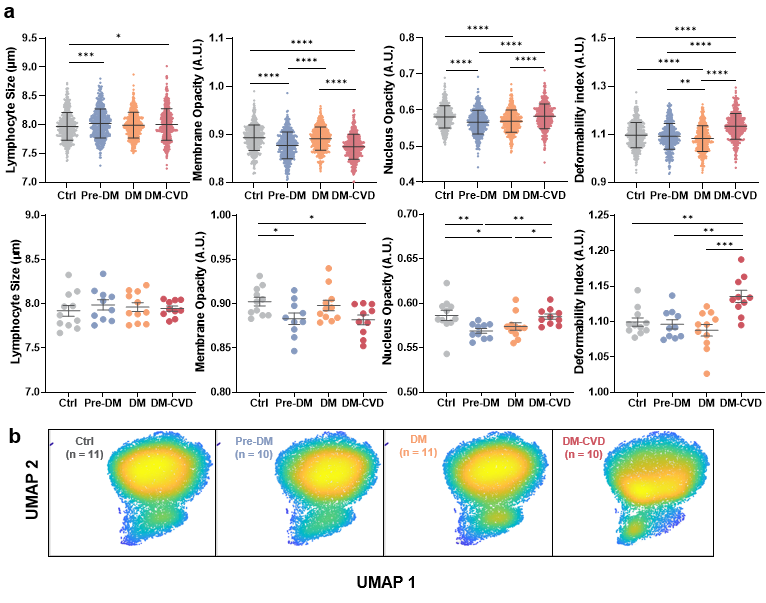


**Fig. S15: Changes in lymphocytes associated with disease severity. a)** Violin plots of single (upper) and dot plots of mean (lower) lymphocytes biophysical properties across donors with different disease severity. **b)** UMAP analysis of impedance detected biophysical properties.

Alteration of biophysical properties in DM-CVD and monocytes

The impedance-based biophysical properties of monocytes are shown in **Fig. S16**a. Cell size exhibited a trend of reduction with disease progression severity. DM-CVD monocytes exhibited the highest nucleus opacity. UMAP analysis of monocytes was also performed where DM-CVD monocytes clustering was partially overlapped with DM monocytes (Fig. S16b).


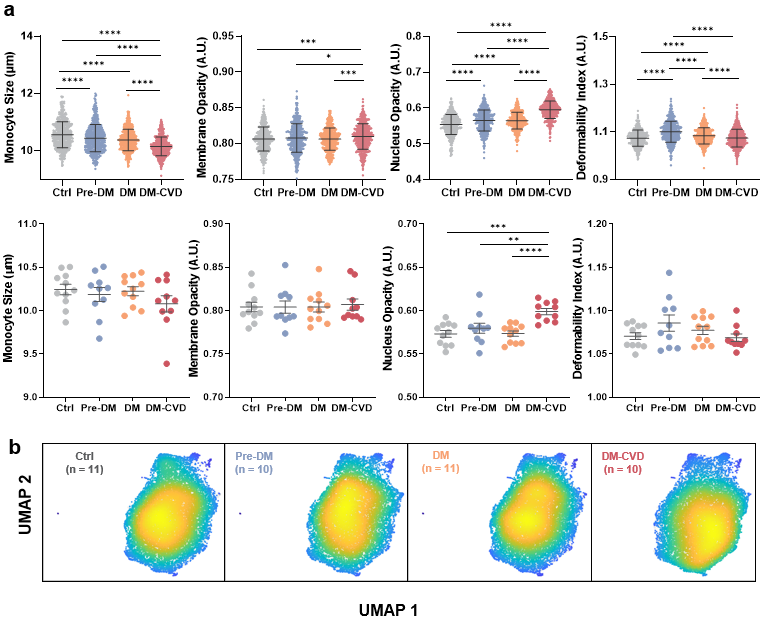


**Fig. S16: Changes in monocytes associated with disease severity. a)** Violin plots of single (upper) and dot plots of mean (lower) monocytes biophysical properties across donors with different disease severity. **b)** UMAP analysis of impedance detected biophysical properties.


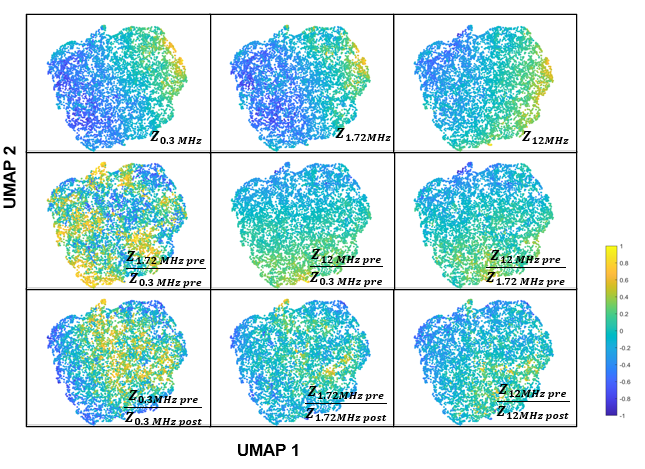


**Fig. S17:** UMAP parameters and magnitude mapping for neutrophils isolated from Ctrl, Pre-DM, DM and DM-CVD subjects.


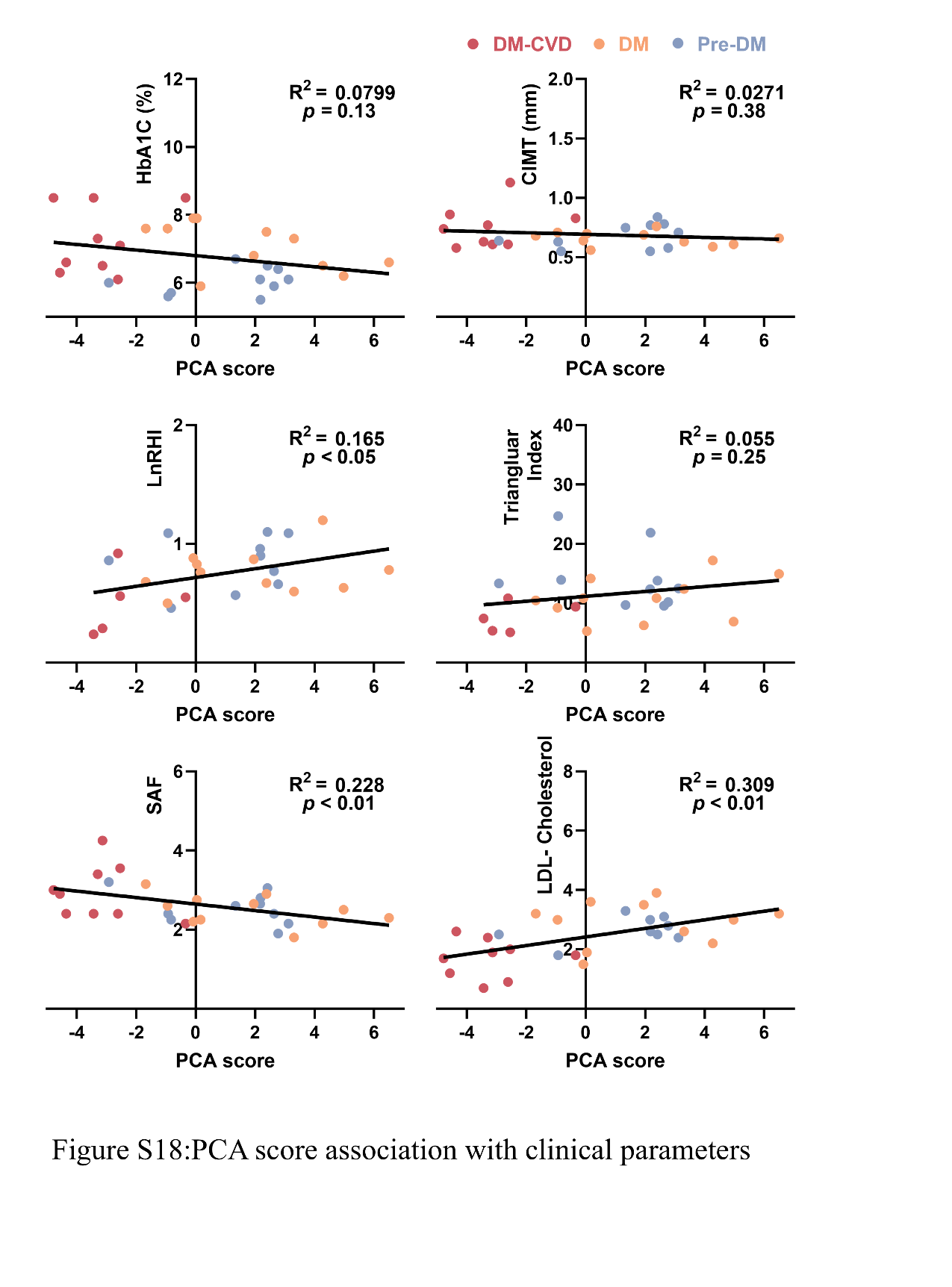


**Fig. S18:** PCA score association with clinical parameters

# Supporting Tables

Table S1: Donor recruitment criteria

| **Healthy**  **(Ctrl)** | **Pre-diabetes**  **(Pre-DM)** | **T2DM**  **(DM)** | **T2DM with CVD**  **(DM-CVD)** |
| --- | --- | --- | --- |
| No T2DM history  No history of CVD complications | Any of the following:   - Fasting glucose 5.9~6.9 mmol/L - 120min glucose 7.8~11 mmol/L - HbA1c 5.8%~6.4%   Without history of CVD complications | Any of the following:   - Fasting glucose ≥7 mmol/L - 120 min glucose ≥11 mmol/L - HbA1c 6.4%~10% (both inclusive)   Without history of CVD complications | Any of the following:   - Fasting glucose ≥7 mmol/L - 120 min glucose ≥11 mmol/L - HbA1c 6.4%~10% (both inclusive)   With history of CVD complications |

Table S2: Clinical characteristics of participants

| **Characteristics** | **Ctrl** | **Pre-DM** | **DM** | **DM-CVD** |
| --- | --- | --- | --- | --- |
| Age (Range) | 24 - 57 | 37 - 67 | 36 - 68 | 48 - 69 |
| Age | 40.13 ± 11.00 | 54.31 ± 8.99 | 51.44 ± 8.80 | 60.40 ± 8.22 |
| BMI | 25.40 ± 9.22 | 27.35 ± 5.29 | 28.28 ± 4.85 | 26.66 ± 5.06 |
| CRP (mg/mL) | 5.43 ± 12.69 | 2.78 ± 3.04 | 2.74 ± 2.53 | 1.54 ± 1.31 |
| HbA1c (%) | 5.36 ± 0.33 | 6.09 ± 0.4 | 7.23 ± 1.85 | 7.2 ± 0.96 |
| Fasting Glucose (mmol/L) | 5.09 ± 0.38 | 5.92 ± 0.66 | 7.46 ± 3.04 | 7.55 ± 1.48 |
| Total Cholesterol (mmol/L) | 5.4 ± 0.71 | 4.66 ± 0.65 | 4.86 ± 1.16 | 3.61 ± 0.77 |
| HDL Cholesterol (mmol/L) | 1.51 ± 0.27 | 1.35 ± 0.38 | 1.23 ± 0.22 | 1.57 ± 0.94 |
| LDL Cholesterol (mmol/L) | 3.49 ± 0.65 | 2.6 ± 0.43 | 2.76 ± 0.69 | 1.68 ± 0.61 |
| Triglyceride (mmol/L) | 0.9 ± 0.32 | 1.81 ± 1.73 | 1.67 ± 0.94 | 1.38 ± 0.76 |
| CIMT | 0.53 ± 0.08 | 0.68 ± 0.12 | 0.67 ± 0.12 | 0.75 ± 0.17 |
| LnRHI | 0.75 ± 0.24 | 0.85 ± 0.23 | 0.75 ± 0.2 | 0.51 ± 0.27 |
| Augmentation Index - SphygmoCor (%) | 36.67 ± 12.56 | 27.08 ± 10.55 | 33.41 ± 9.87 | 24.56 ± 12.43 |
| PWV (m/s) | 6.07 ± 0.72 | 7.31 ± 0.87 | 8.17 ± 1.58 | 8.3 ± 1.80 |
| Skin Autofluorescence (A.U.) | 2.31 ± 0.5 | 2.56 ± 0.5 | 2.64 ± 0.52 | 2.96 ± 0.65 |

*Average value shown with SD, unless otherwise indicated.*

Reference

[1] H. M. Tay, W. H. Yeap, R. Dalan, S. C. Wong, H. W. Hou, *Analytical Chemistry* **2018**, 90, 14535.

[2] C. Petchakup, H. Yang, L. Gong, L. He, H. M. Tay, R. Dalan, A. J. Chung, K. H. H. Li, H. W. Hou, *Small* **2022**, 18, 2104822.

[3] L. He, J. Tan, S. Y. Ng, K. H. H. Li, J. Han, S. Y. Chew, H. W. Hou, *Advanced Materials Technologies* **2024**, 9, 2400589.

[4] M. I. Love, W. Huber, S. Anders, *Genome Biol* **2014**, 15, 550.

[5] A. Subramanian, P. Tamayo, V. K. Mootha, S. Mukherjee, B. L. Ebert, M. A. Gillette, A. Paulovich, S. L. Pomeroy, T. R. Golub, E. S. Lander, J. P. Mesirov, *Proc Natl Acad Sci U S A* **2005**, 102, 15545.

[6] G. Yu, L. G. Wang, G. R. Yan, Q. Y. He, *Bioinformatics* **2015**, 31, 608.

[7] M. Urbanska, H. E. Muñoz, J. Shaw Bagnall, O. Otto, S. R. Manalis, D. Di Carlo, J. Guck, *Nature Methods* **2020**, 17, 587.

[8] Chii J. Chan, Andrew E. Ekpenyong, S. Golfier, W. Li, Kevin J. Chalut, O. Otto, J. Elgeti, J. Guck, F. Lautenschläger, *Biophysical Journal* **2015**, 108, 1856.
